# Supplementary figures and images for: Basis of single-seed formation in chestnut: cytomorphological observations reveal ovule developmental patterns of Castanea henryi
Source: PeerJ. 2025 Jan 2;13:e18711. doi: 10.7717/peerj.18711 (PMC11700494; doi:10.7717/peerj.18711)

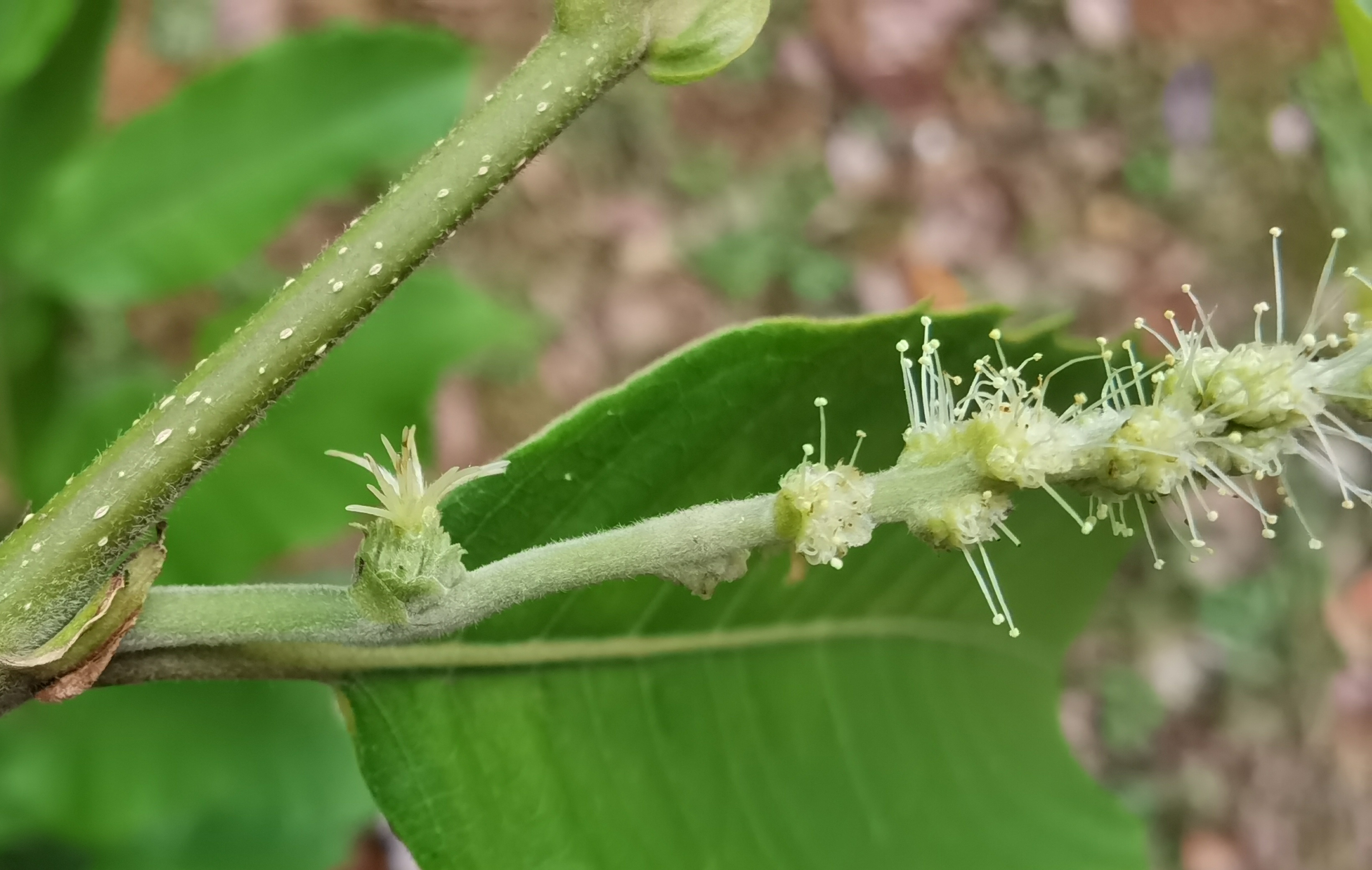

Supplement: Supplemental Information 1 [file peerj-13-18711-s001.zip › 1-a.jpg]

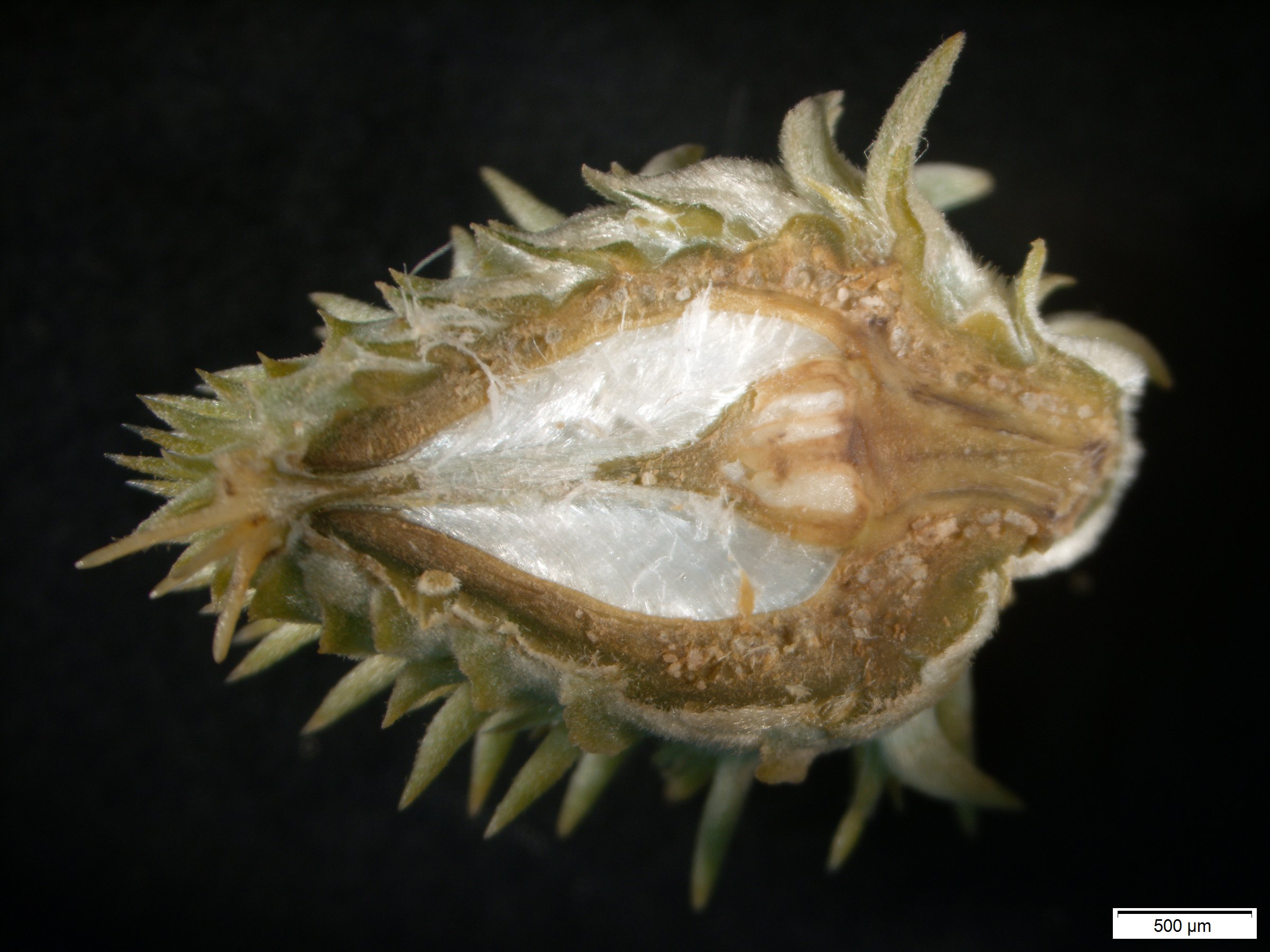

Supplement: Supplemental Information 1 [file peerj-13-18711-s001.zip › 1-b.jpg]

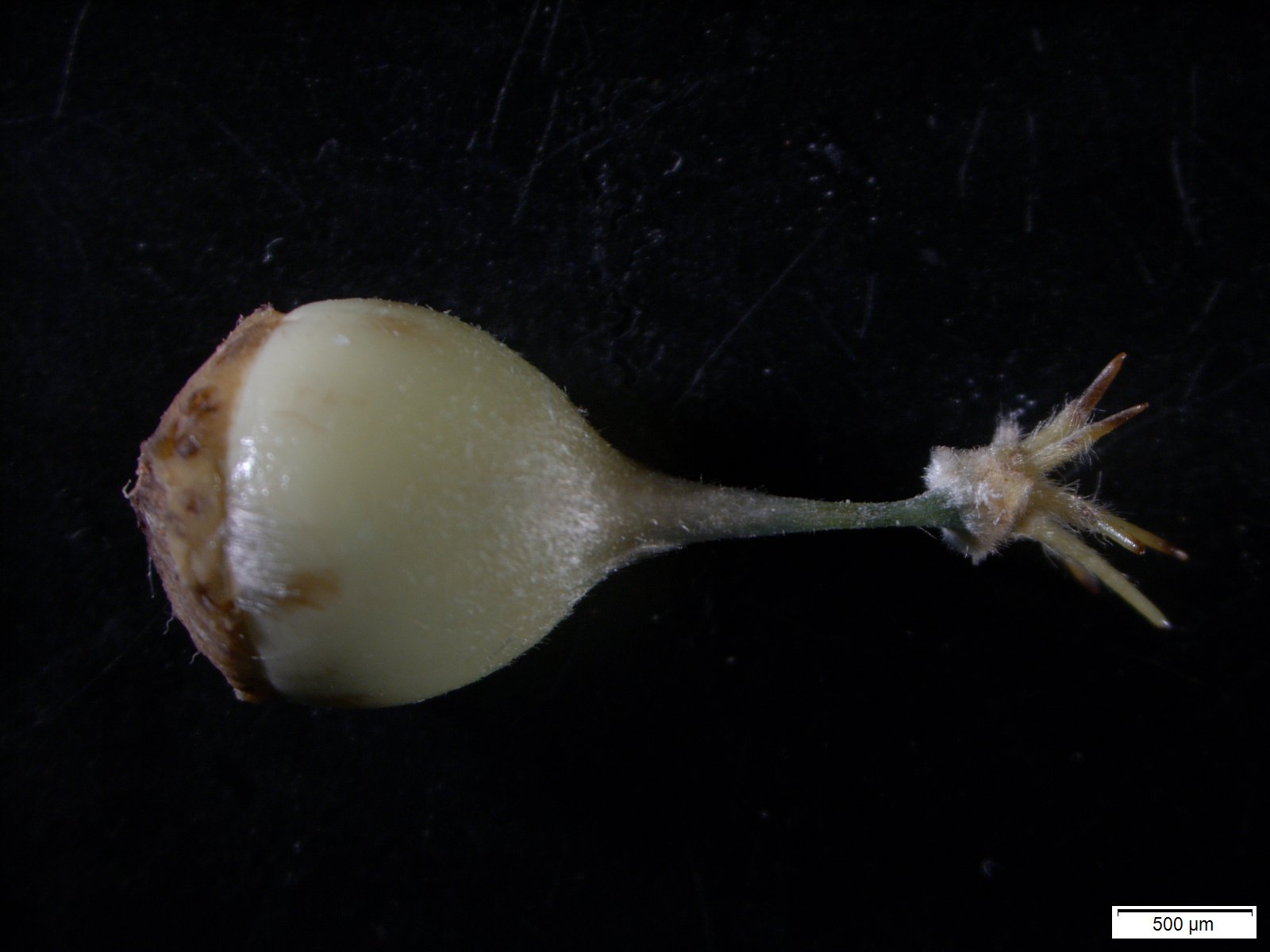

Supplement: Supplemental Information 1 [file peerj-13-18711-s001.zip › 1-c.jpg]

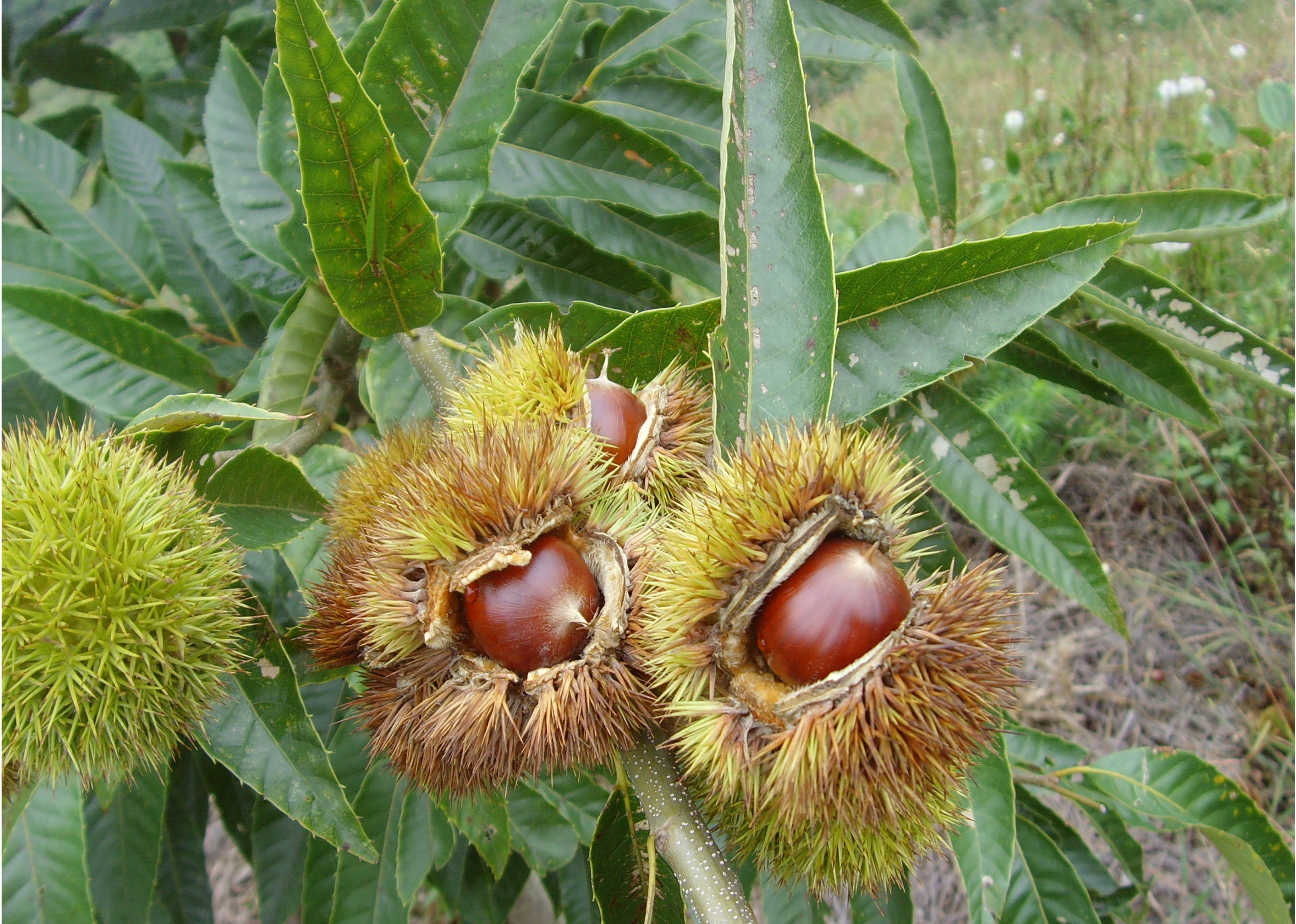

Supplement: Supplemental Information 1 [file peerj-13-18711-s001.zip › 1-d.jpg]

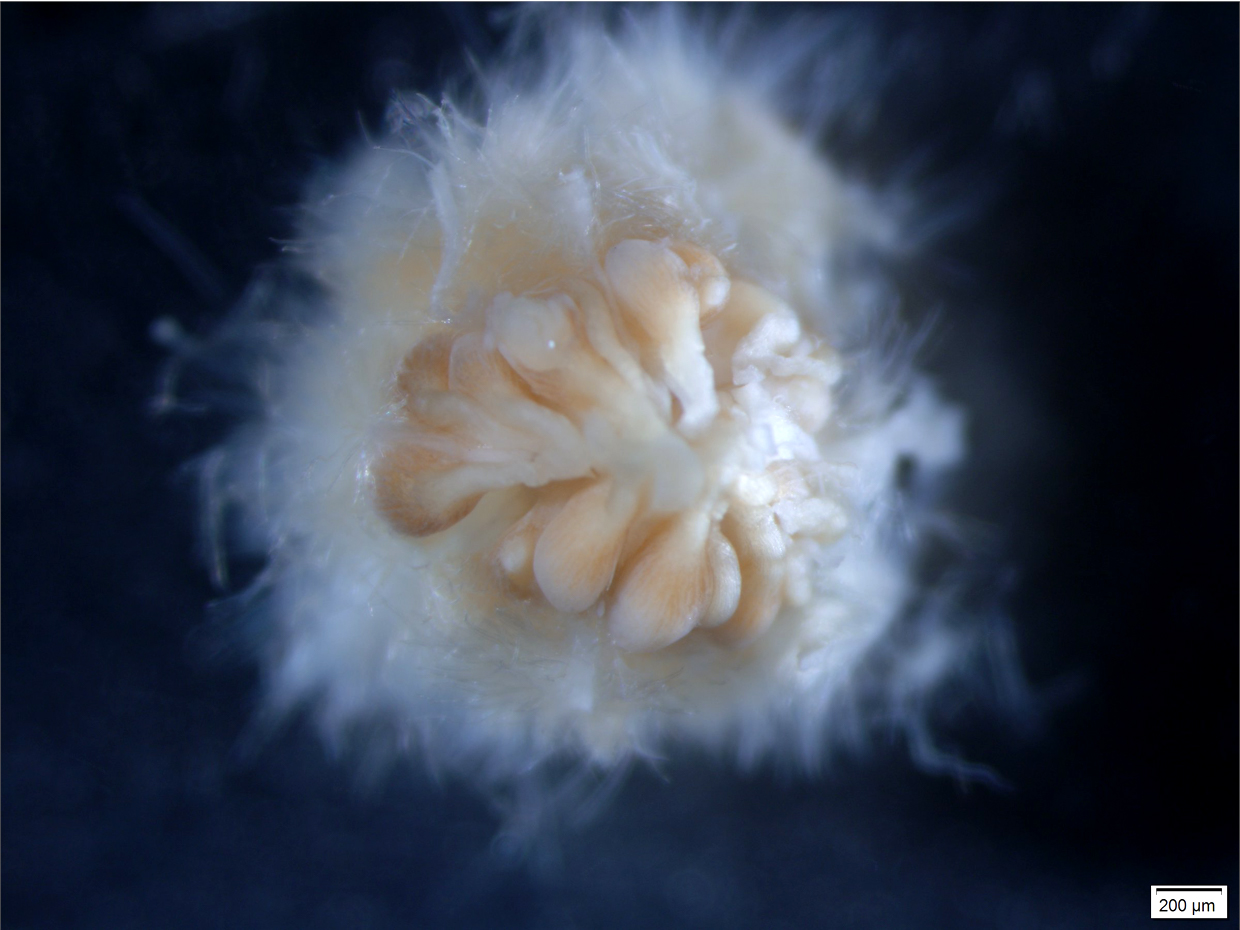

Supplement: Supplemental Information 2 [file peerj-13-18711-s002.zip › 2--g.jpg]

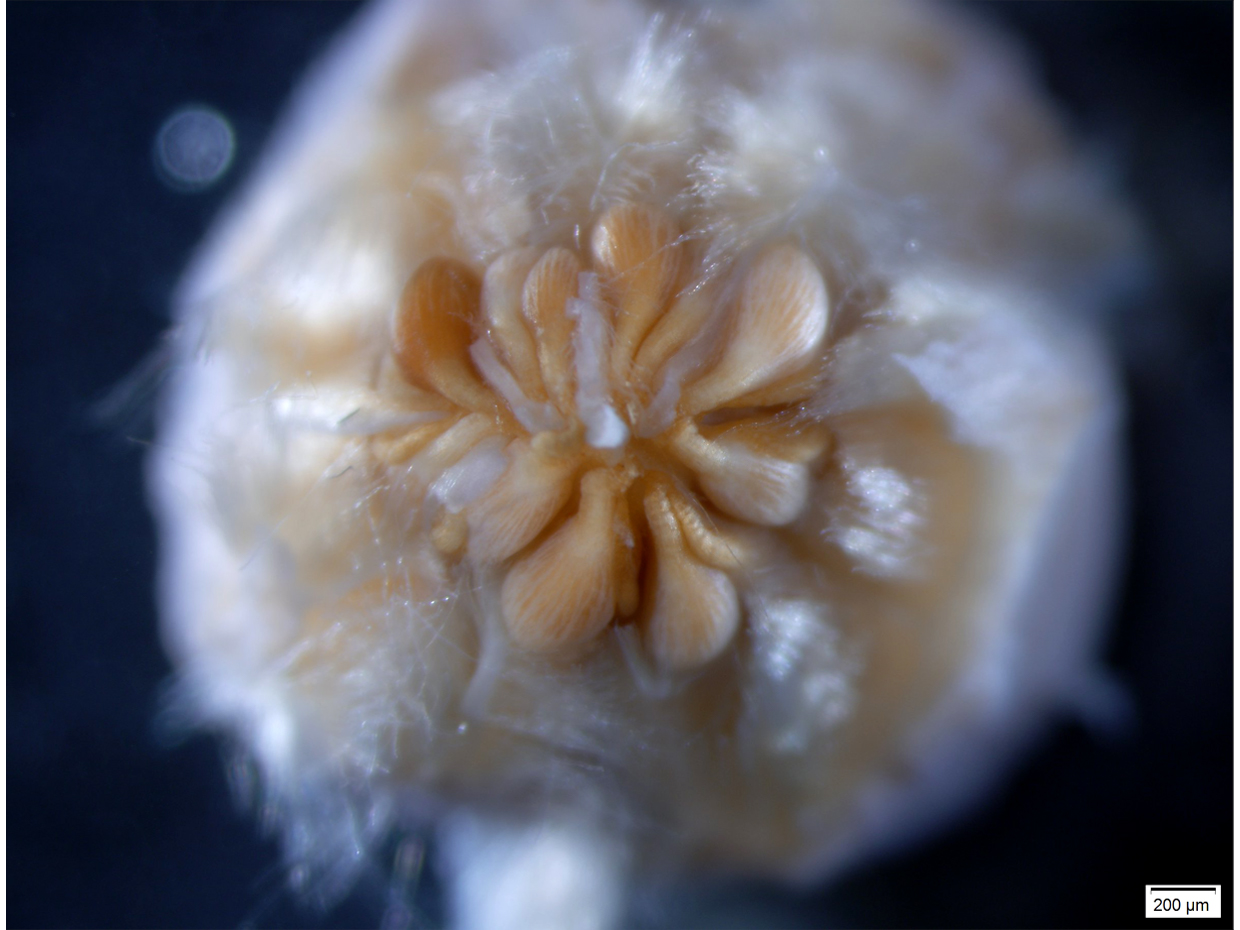

Supplement: Supplemental Information 2 [file peerj-13-18711-s002.zip › 2--h.jpg]

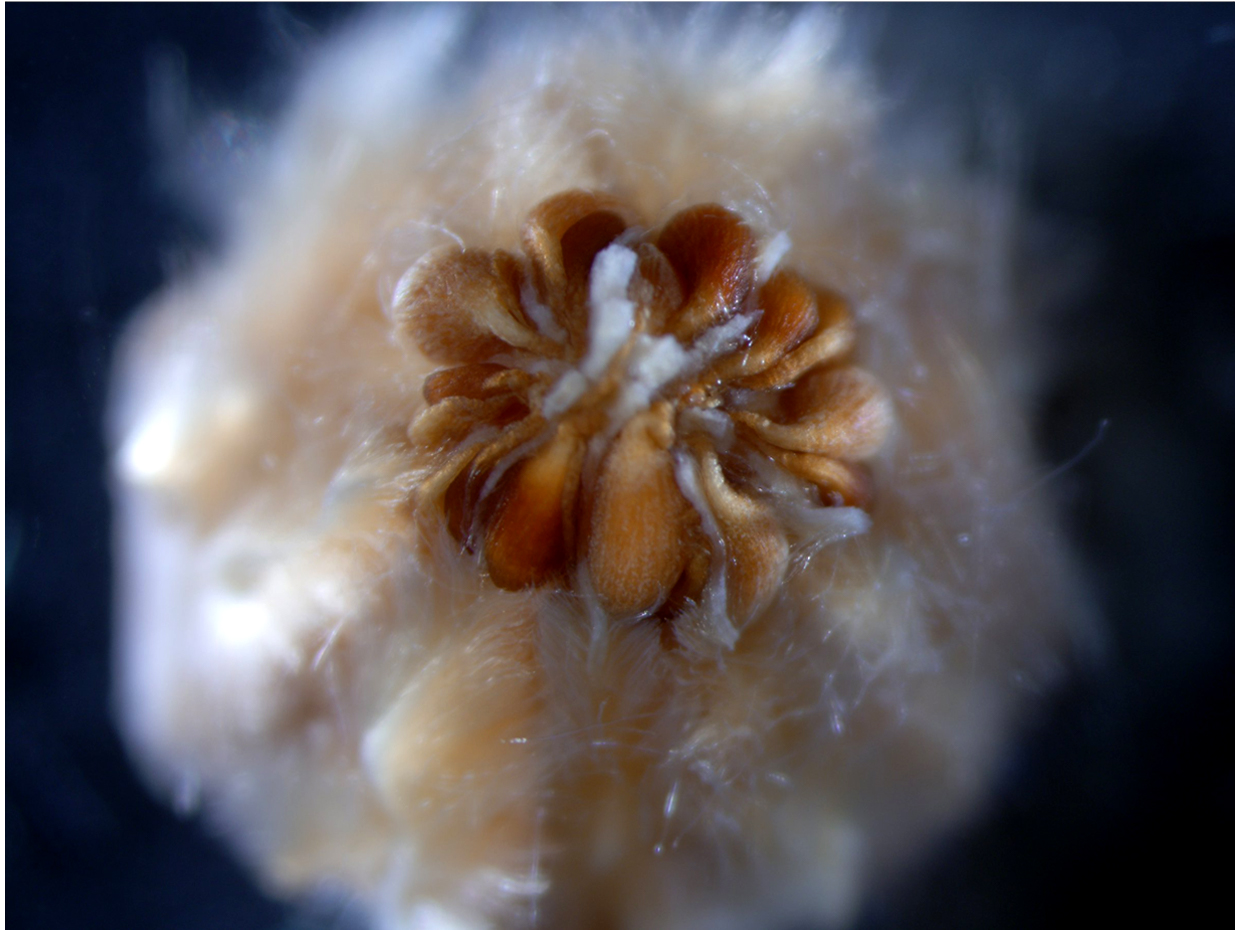

Supplement: Supplemental Information 2 [file peerj-13-18711-s002.zip › 2--i.jpg]

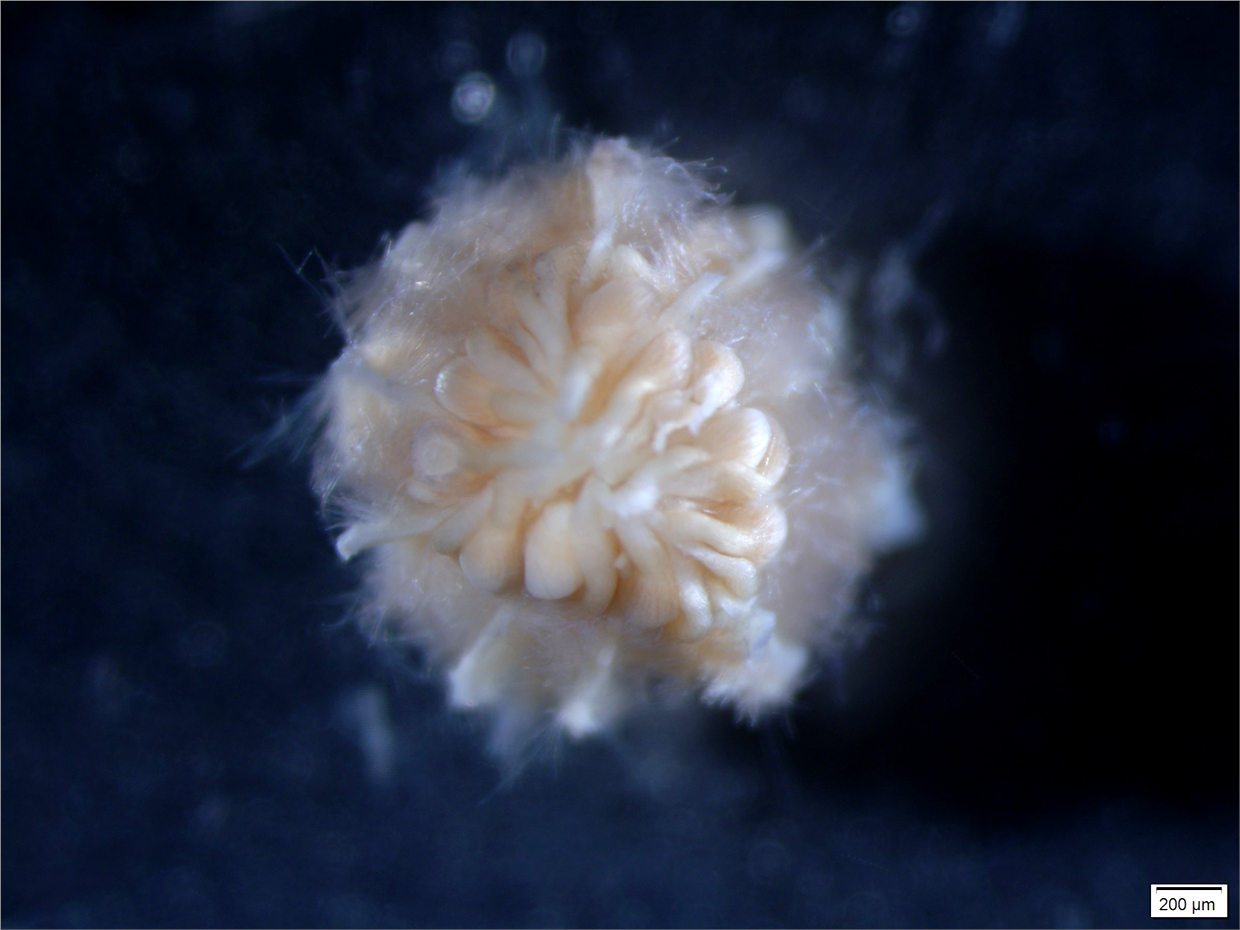

Supplement: Supplemental Information 2 [file peerj-13-18711-s002.zip › 2-a.jpg]

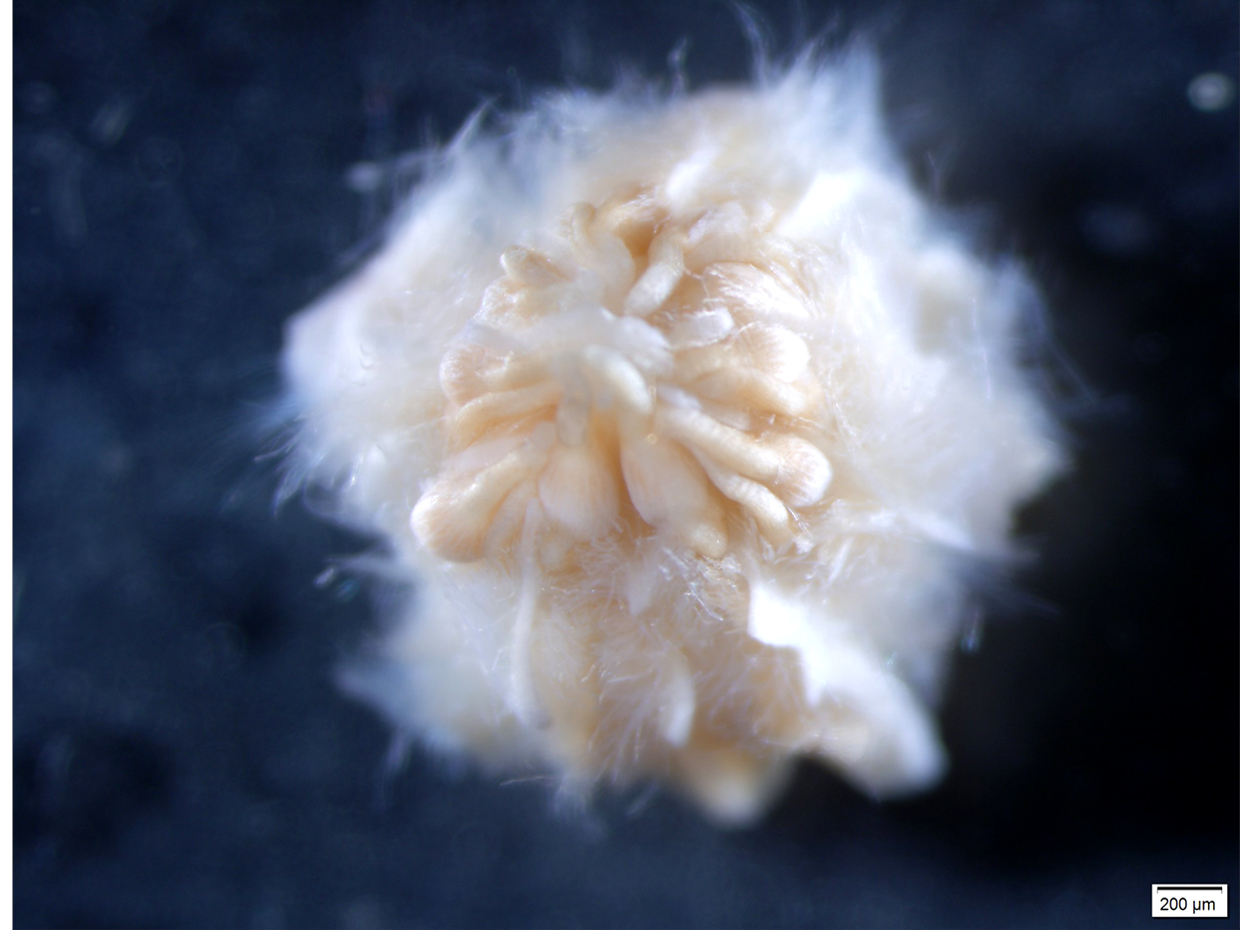

Supplement: Supplemental Information 2 [file peerj-13-18711-s002.zip › 2-b.jpg]

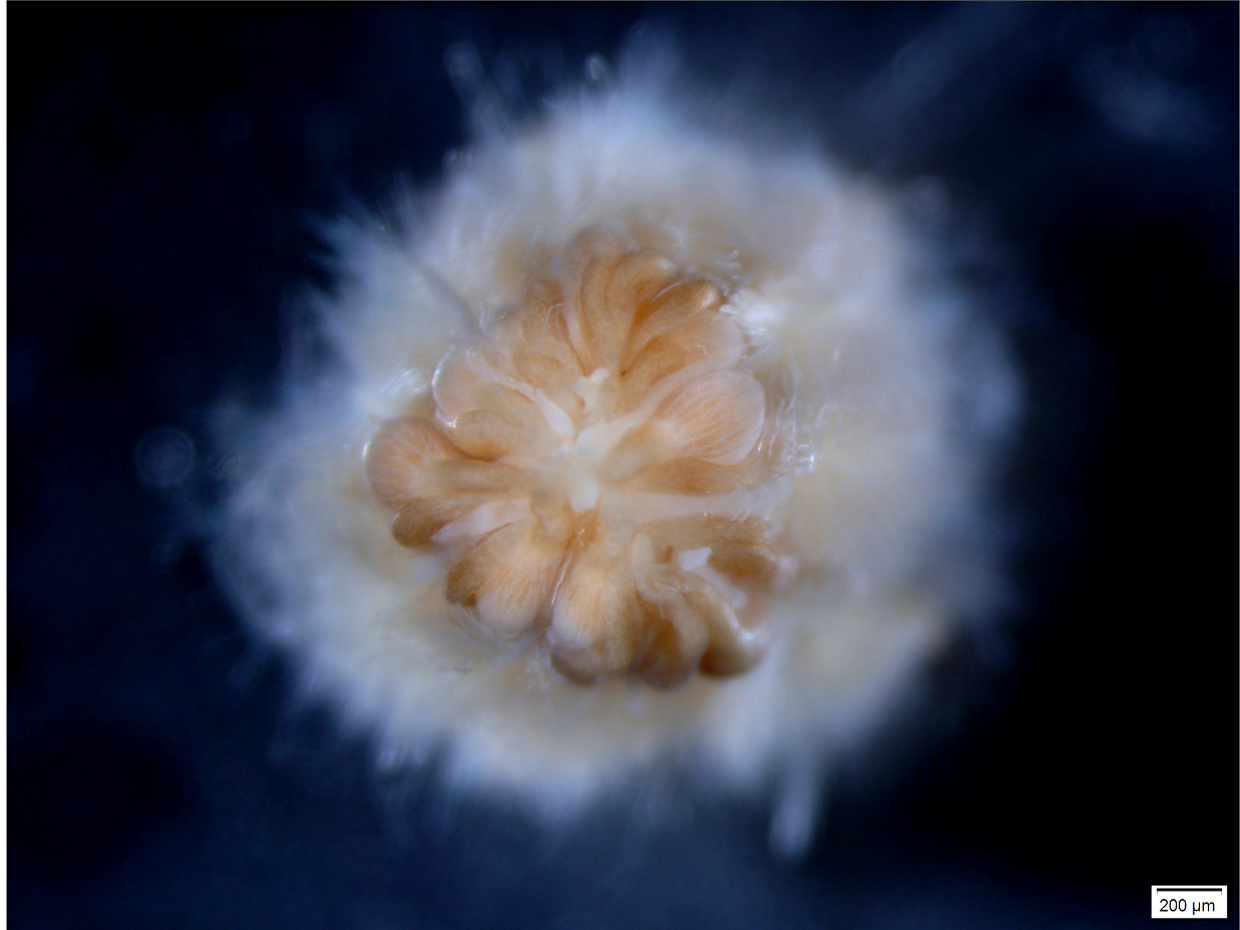

Supplement: Supplemental Information 2 [file peerj-13-18711-s002.zip › 2-c.jpg]

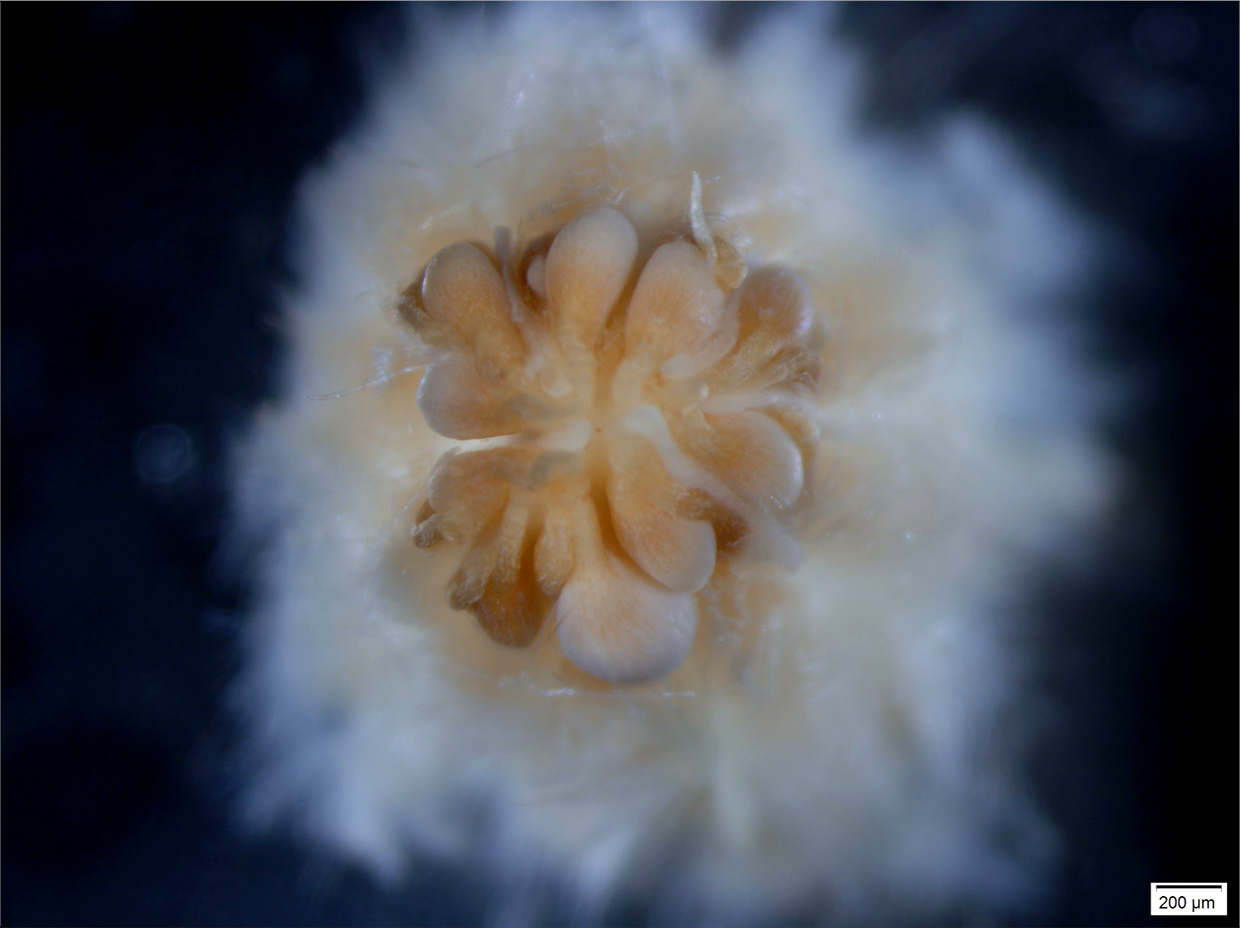

Supplement: Supplemental Information 2 [file peerj-13-18711-s002.zip › 2-d.jpg]

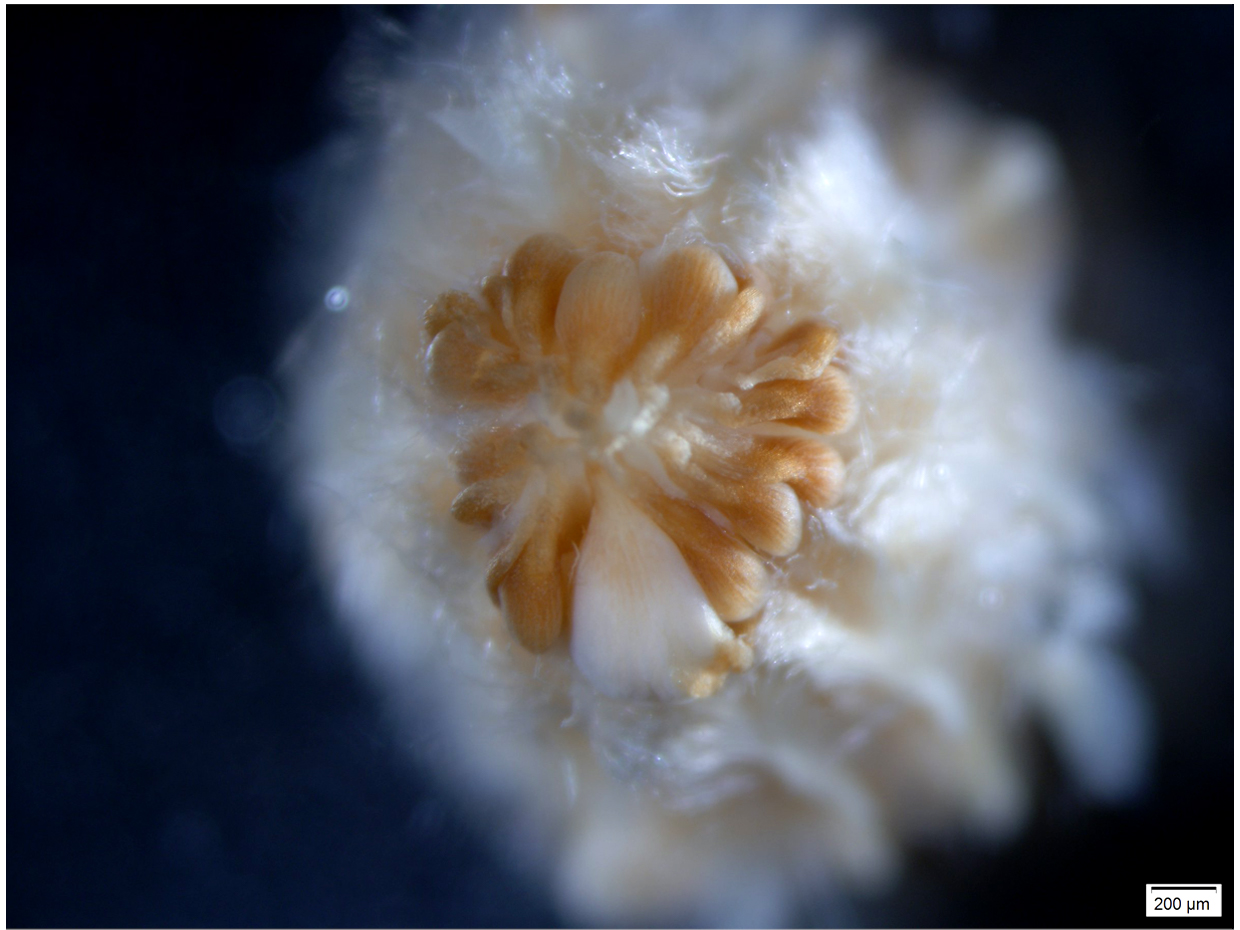

Supplement: Supplemental Information 2 [file peerj-13-18711-s002.zip › 2-e.jpg]

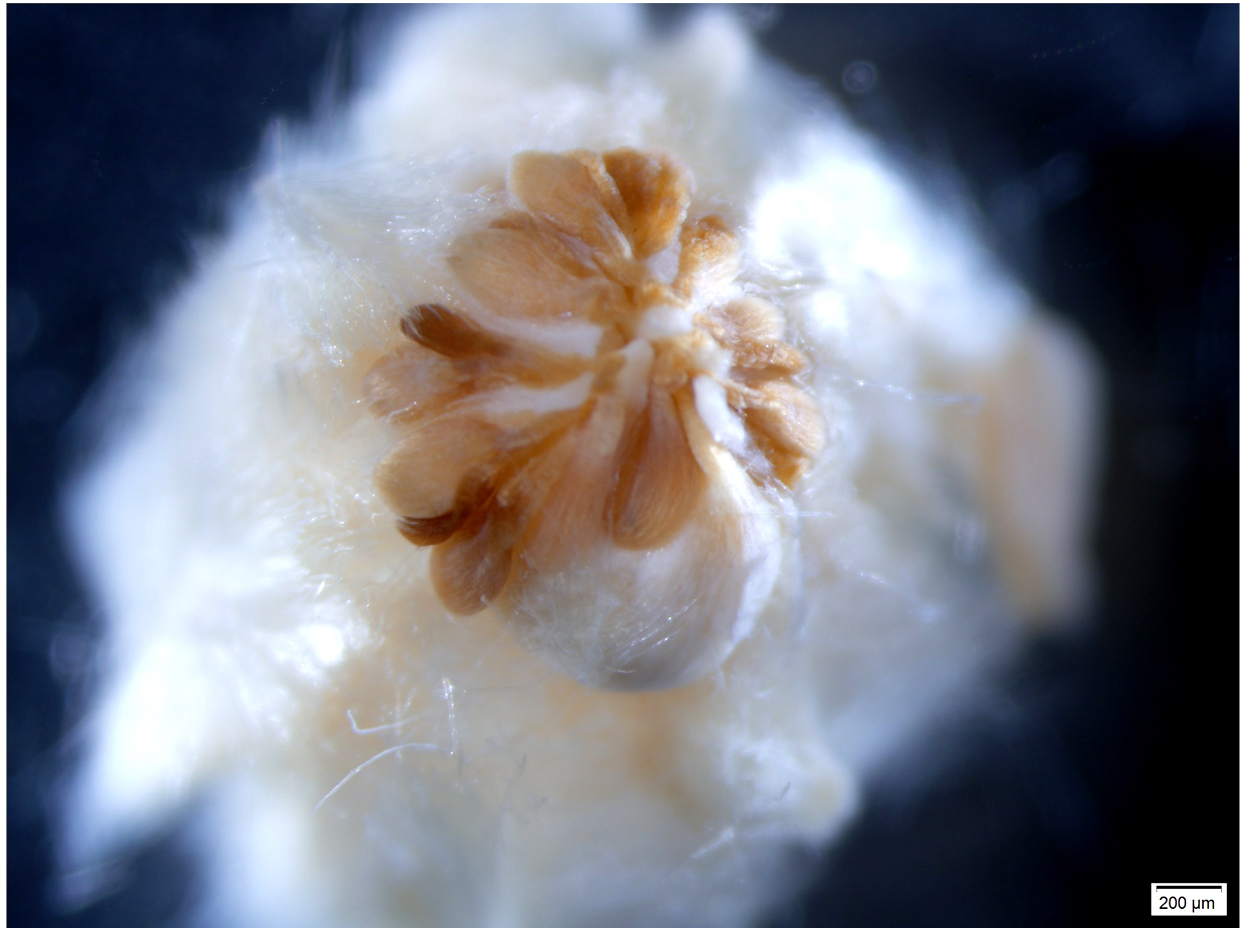

Supplement: Supplemental Information 2 [file peerj-13-18711-s002.zip › 2-f.jpg]

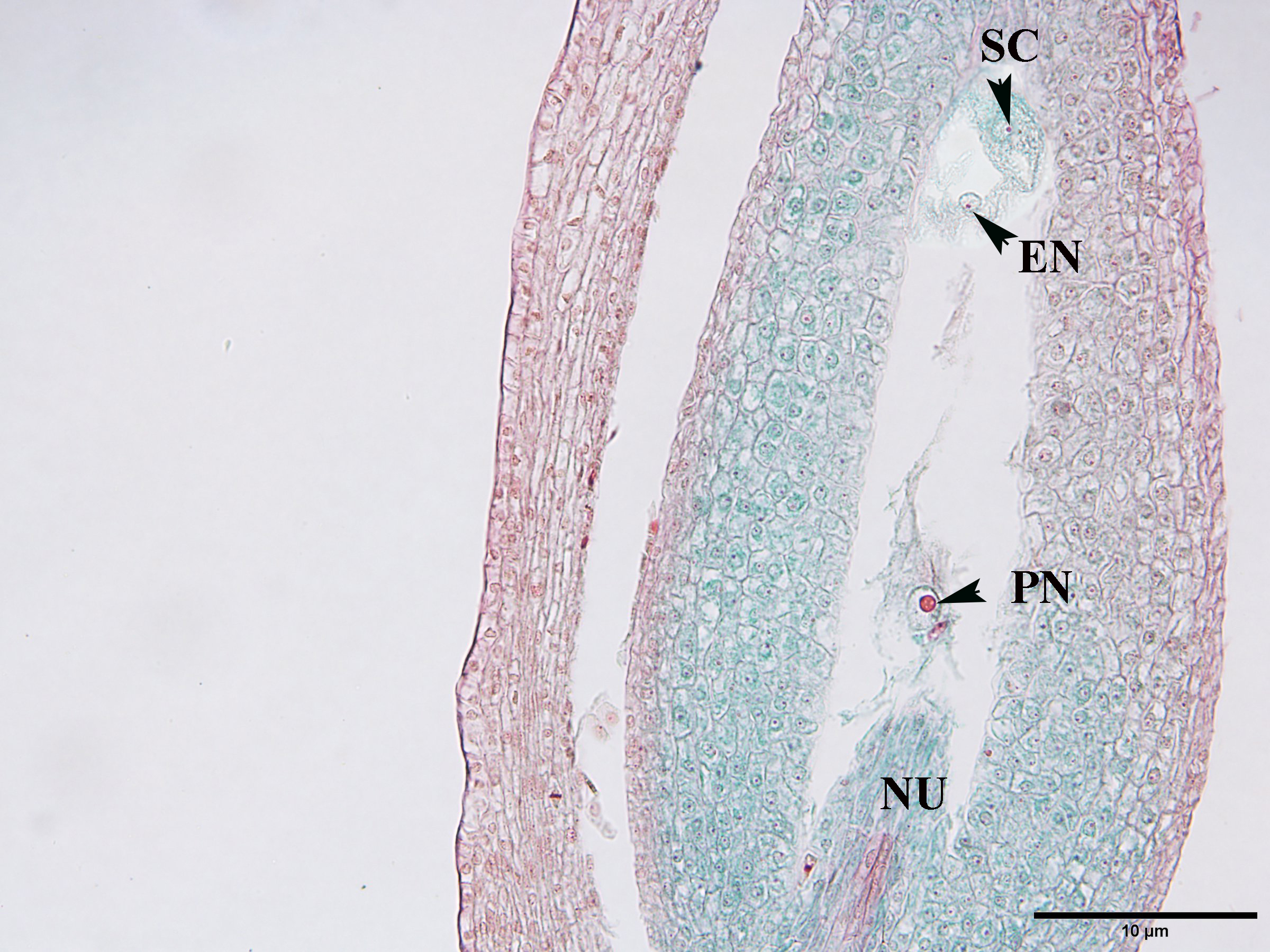

Supplement: Supplemental Information 3 [file peerj-13-18711-s003.zip › 3-a.jpg]

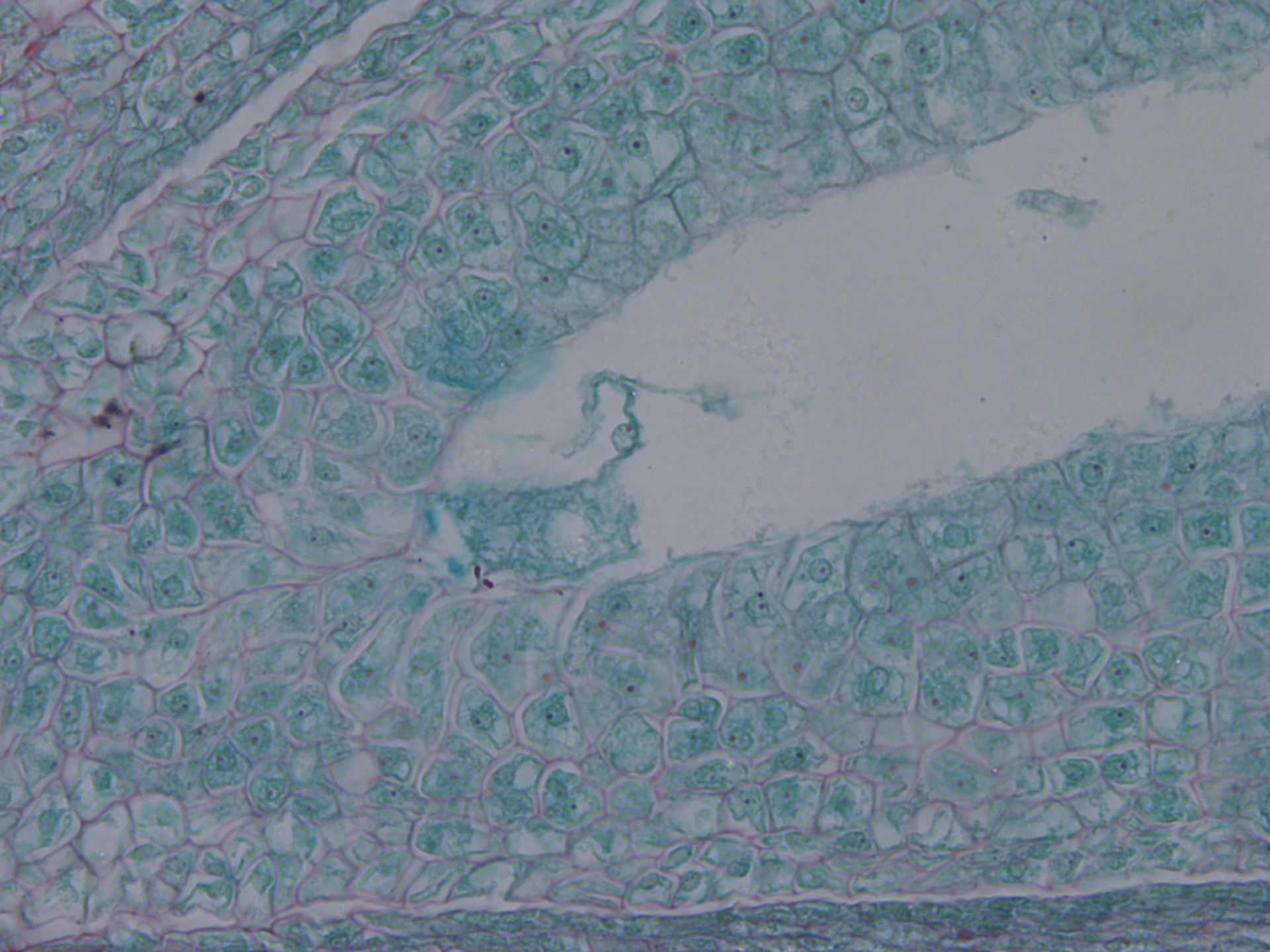

Supplement: Supplemental Information 3 [file peerj-13-18711-s003.zip › 3-b.jpg]

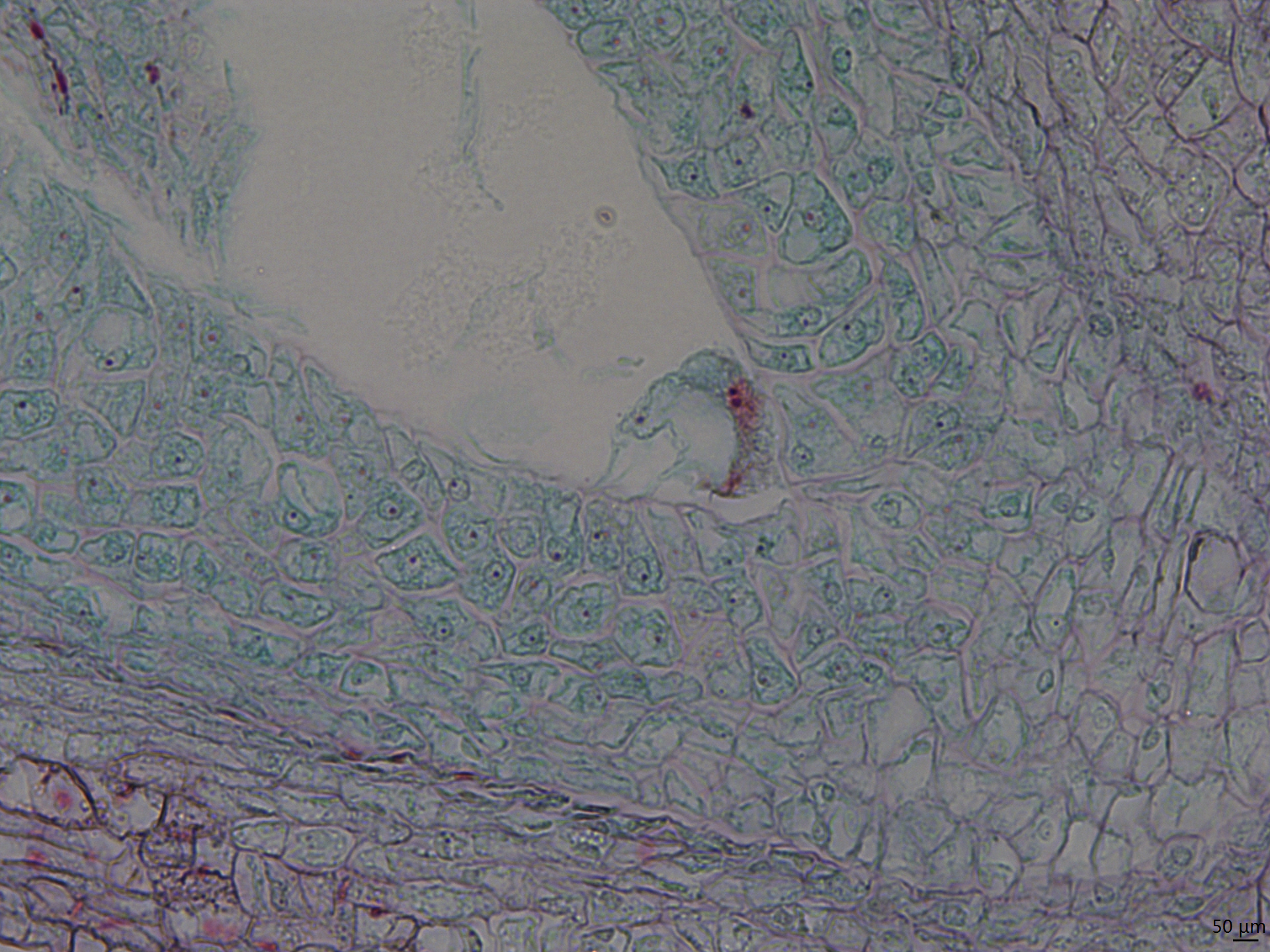

Supplement: Supplemental Information 3 [file peerj-13-18711-s003.zip › 3-c.jpg]

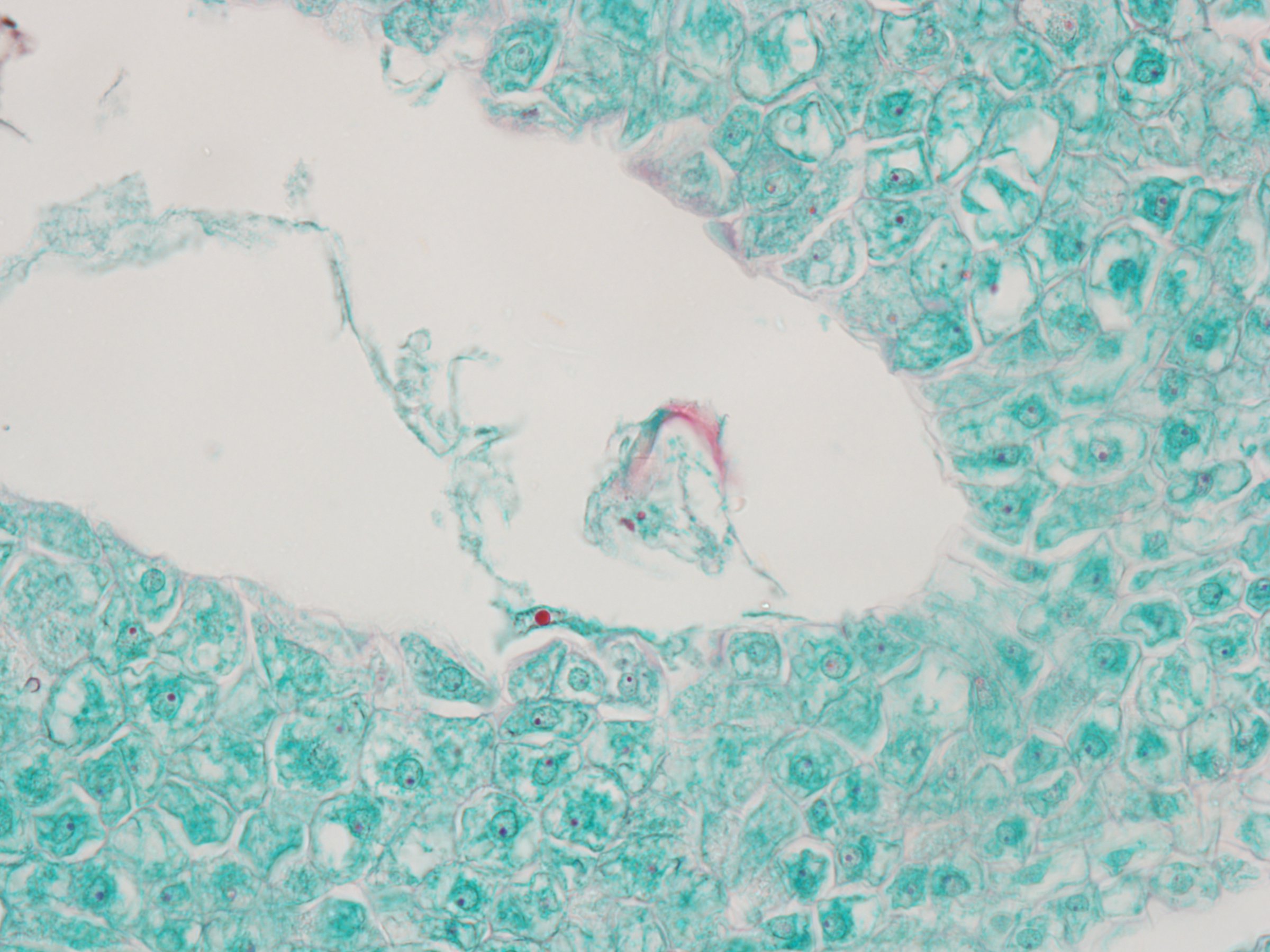

Supplement: Supplemental Information 3 [file peerj-13-18711-s003.zip › 3-d.jpg]

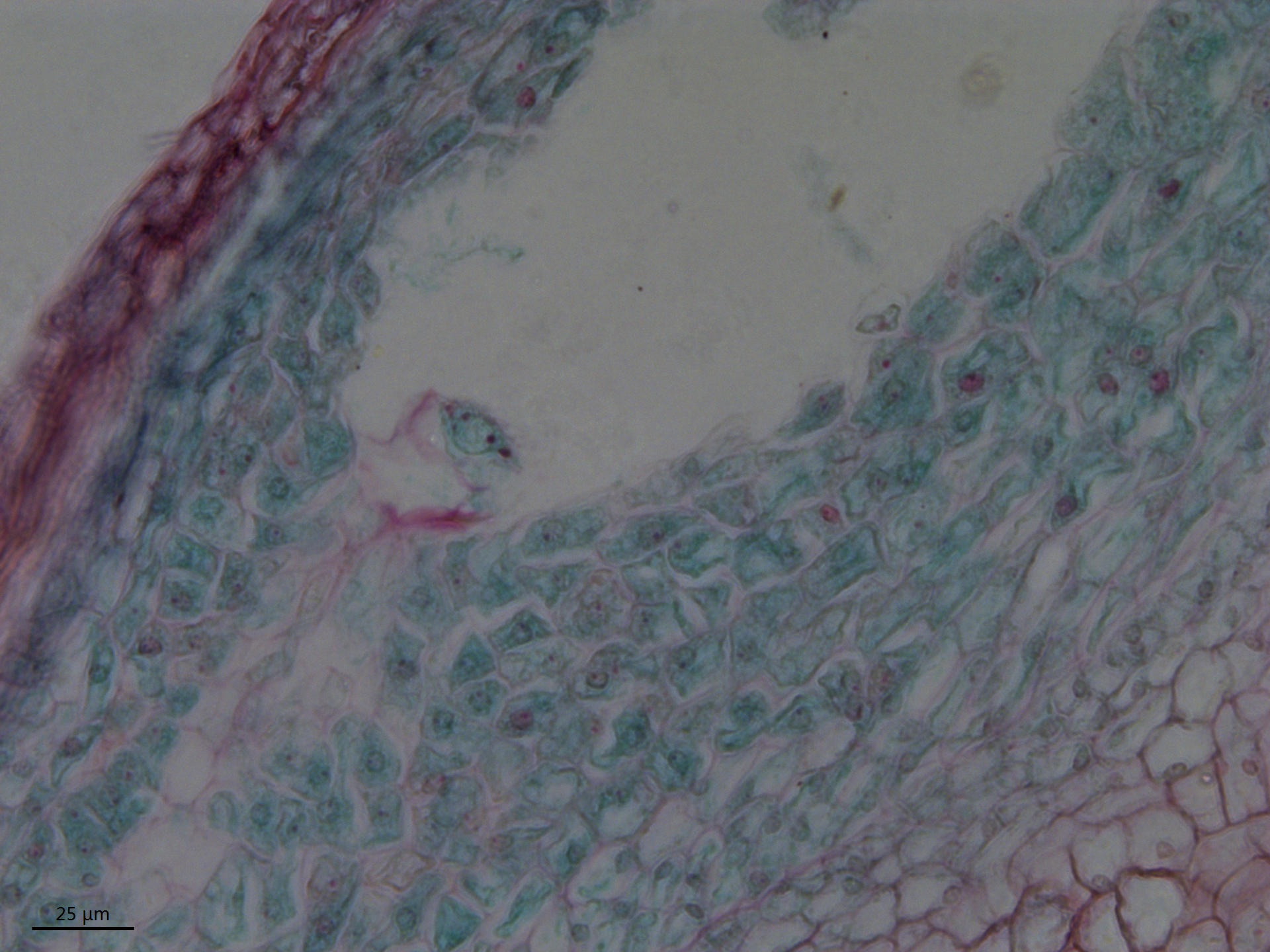

Supplement: Supplemental Information 3 [file peerj-13-18711-s003.zip › 3-e.tif]

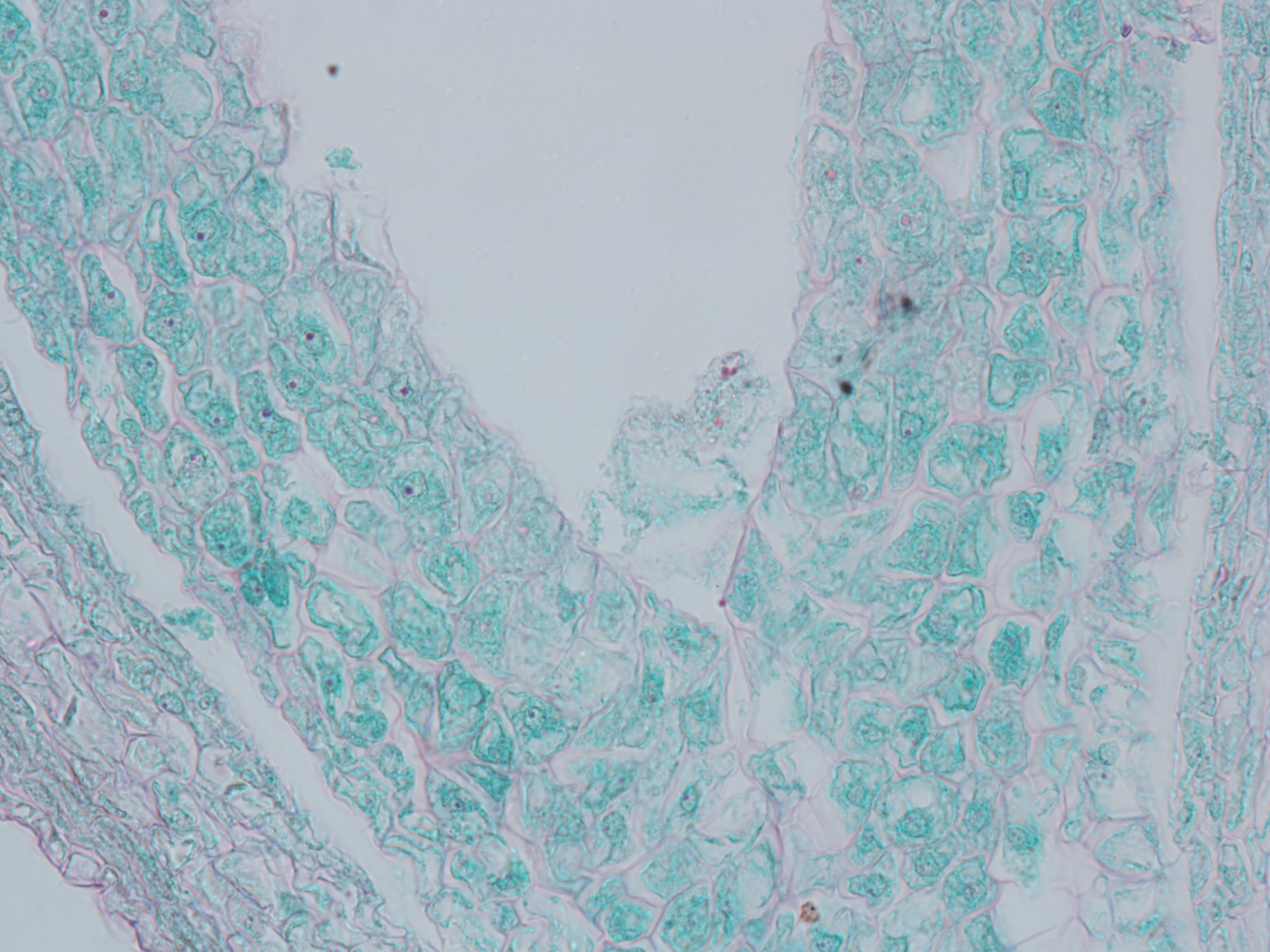

Supplement: Supplemental Information 3 [file peerj-13-18711-s003.zip › 3-f.jpg]

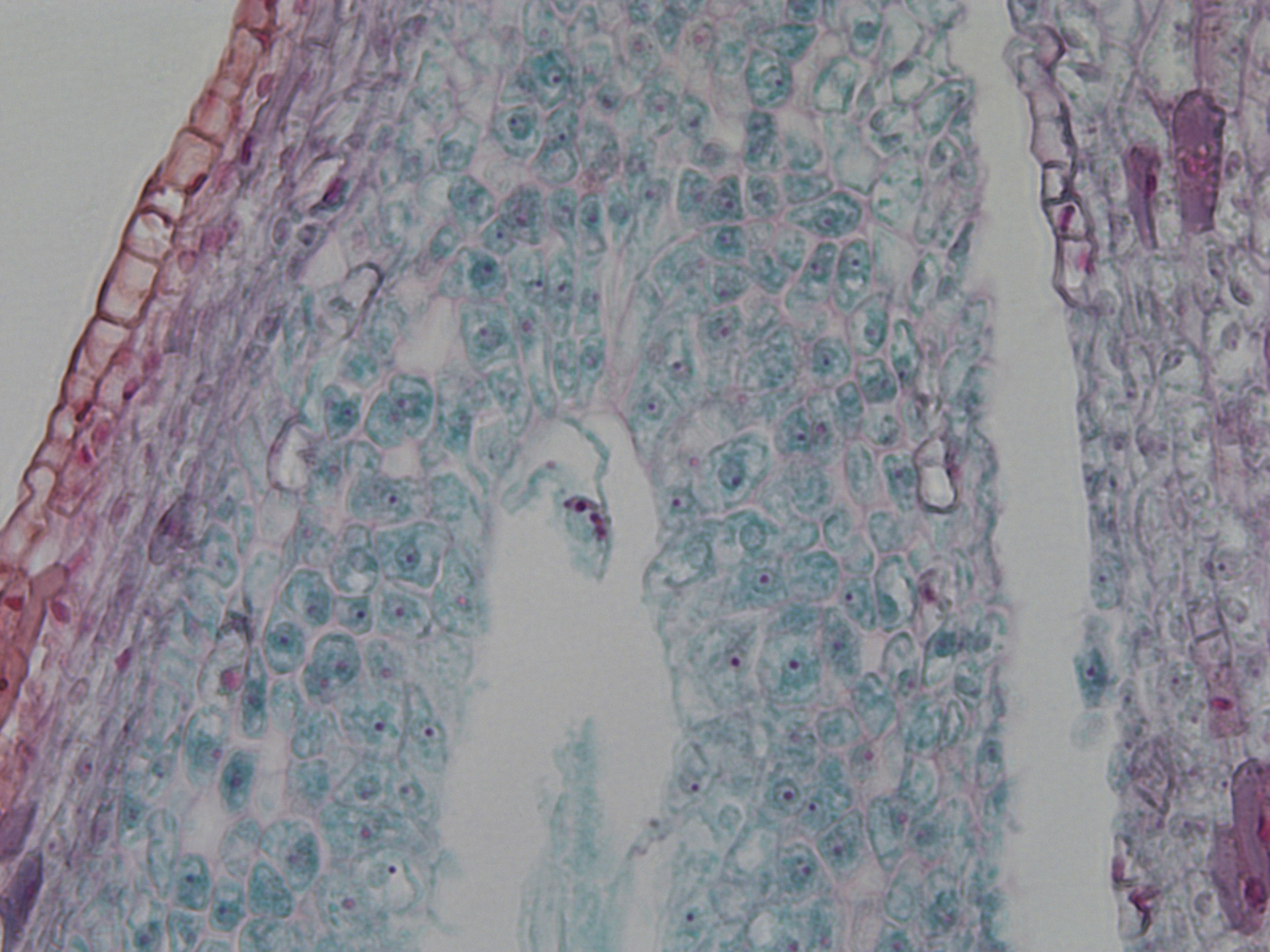

Supplement: Supplemental Information 3 [file peerj-13-18711-s003.zip › 3-g.jpg]

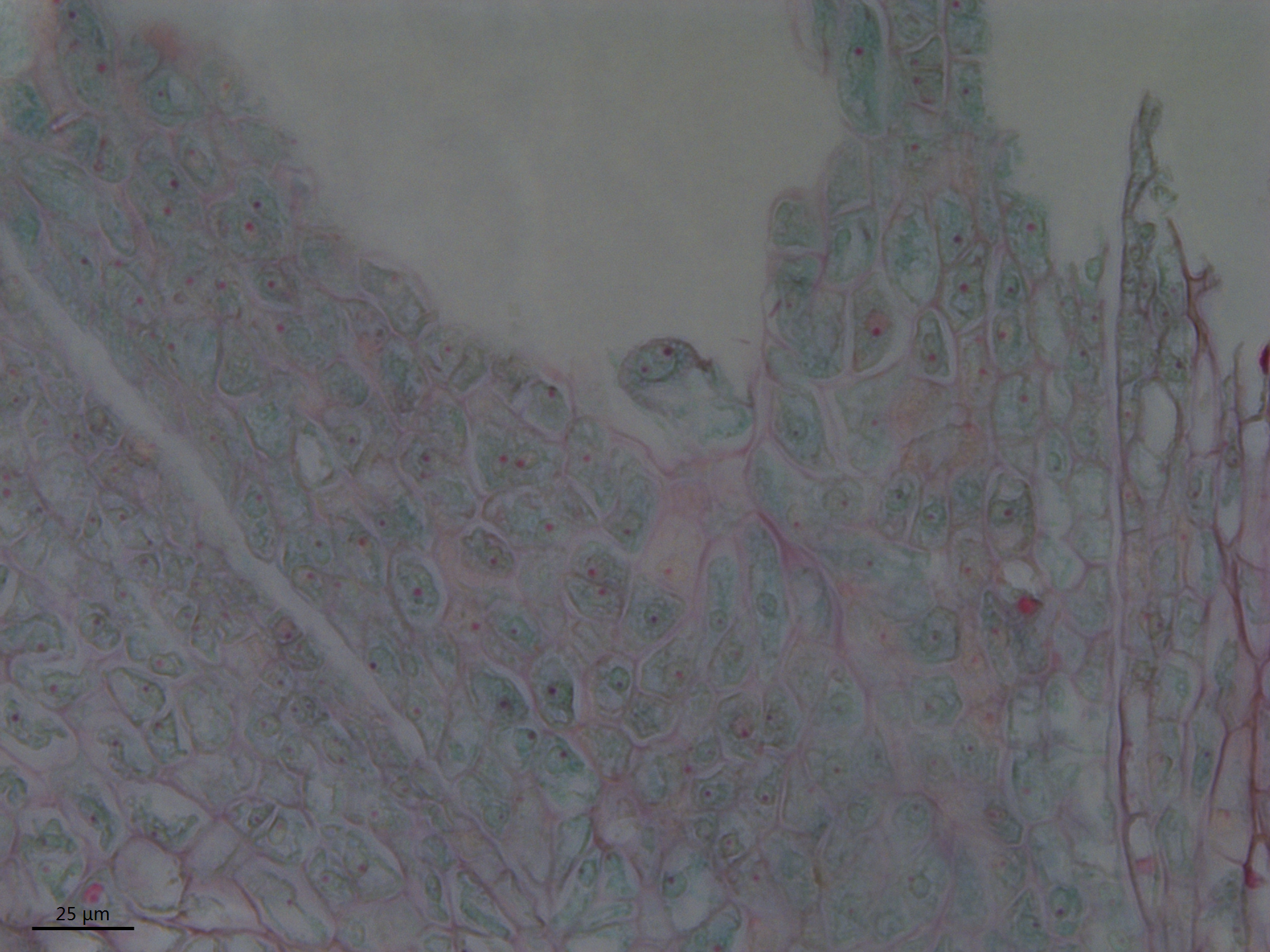

Supplement: Supplemental Information 3 [file peerj-13-18711-s003.zip › 3-h.tif]

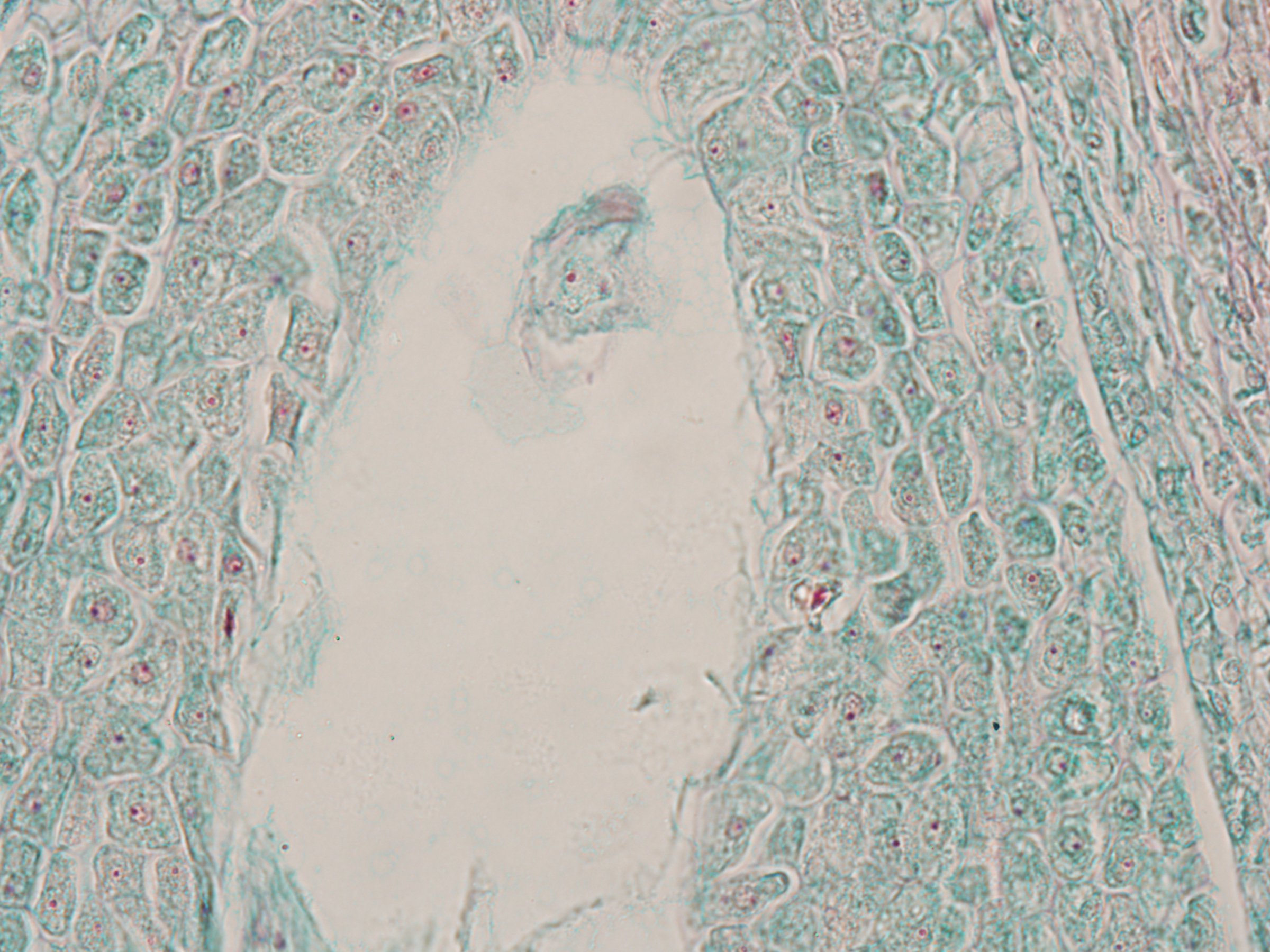

Supplement: Supplemental Information 3 [file peerj-13-18711-s003.zip › 3-i.jpg]

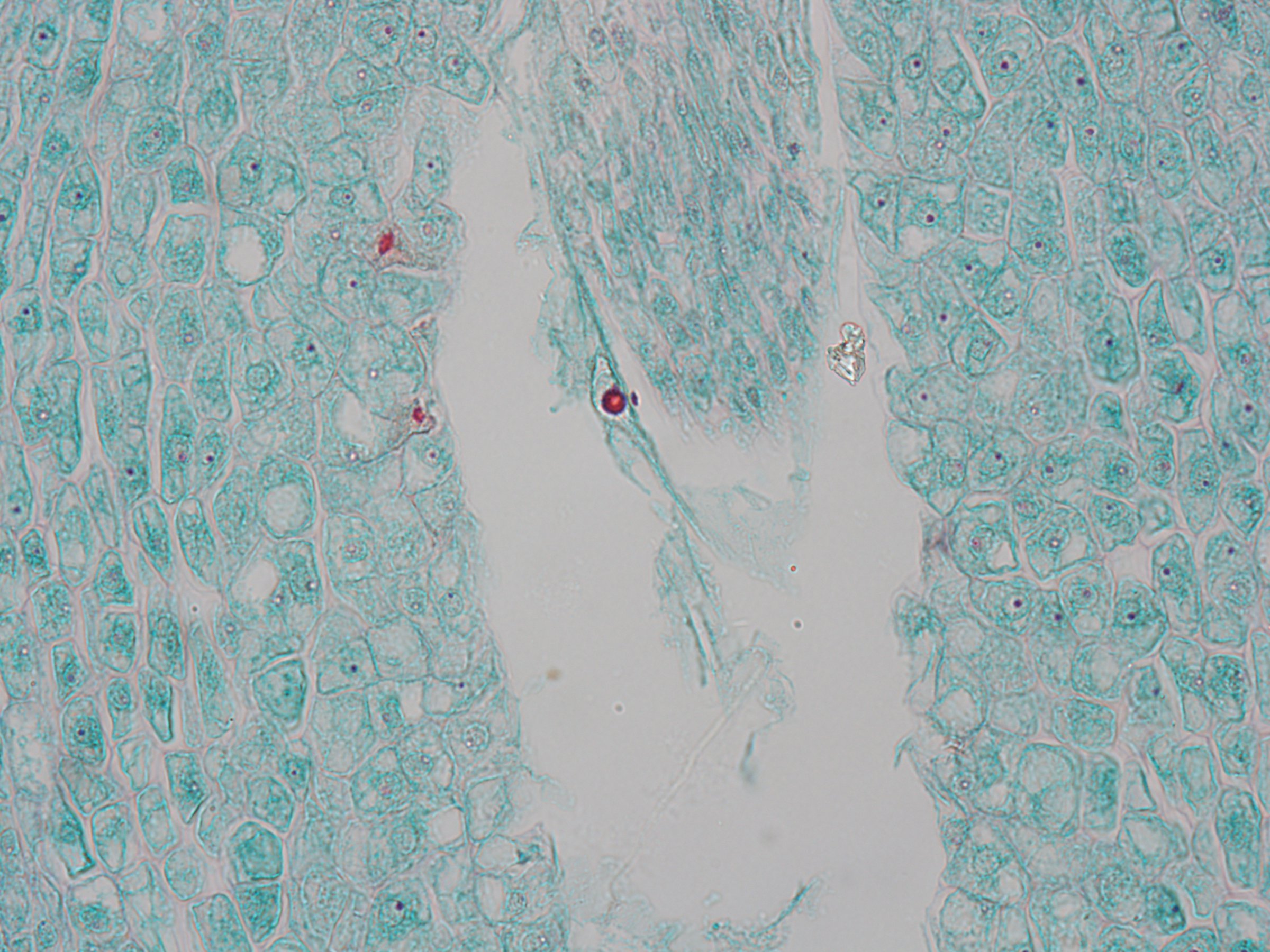

Supplement: Supplemental Information 3 [file peerj-13-18711-s003.zip › 3-j.jpg]

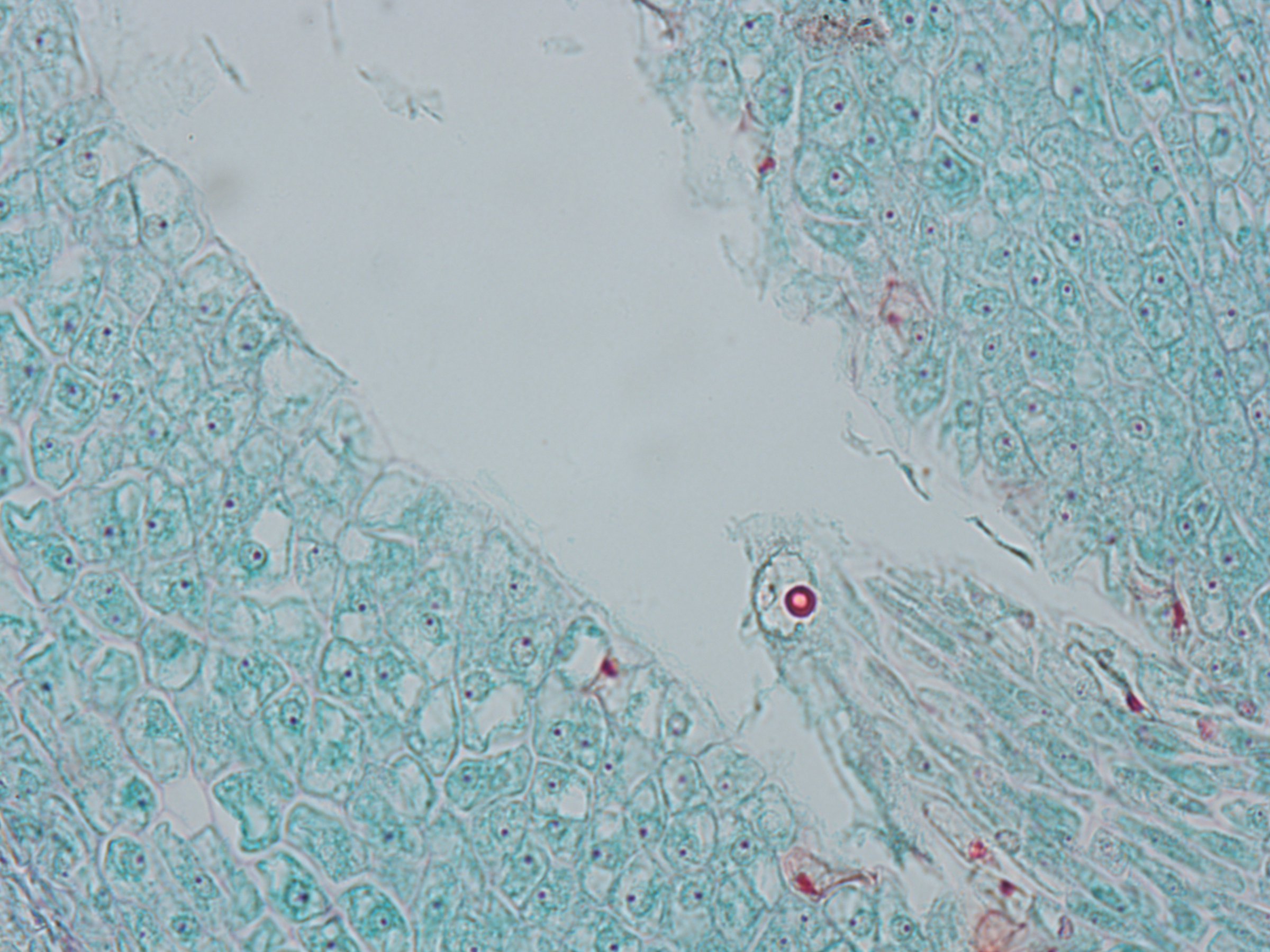

Supplement: Supplemental Information 3 [file peerj-13-18711-s003.zip › 3-k.jpg]

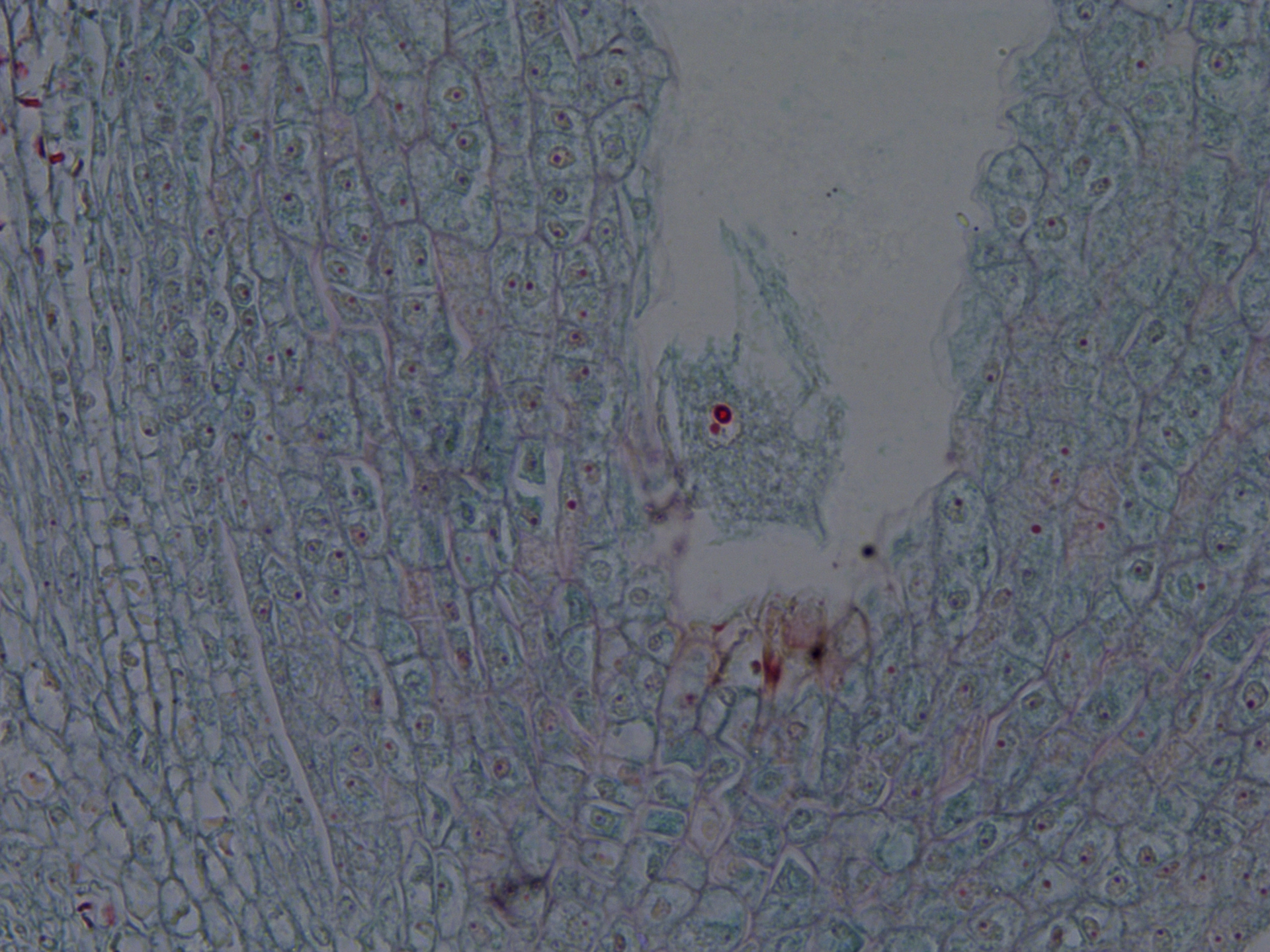

Supplement: Supplemental Information 3 [file peerj-13-18711-s003.zip › 3-l.jpg]

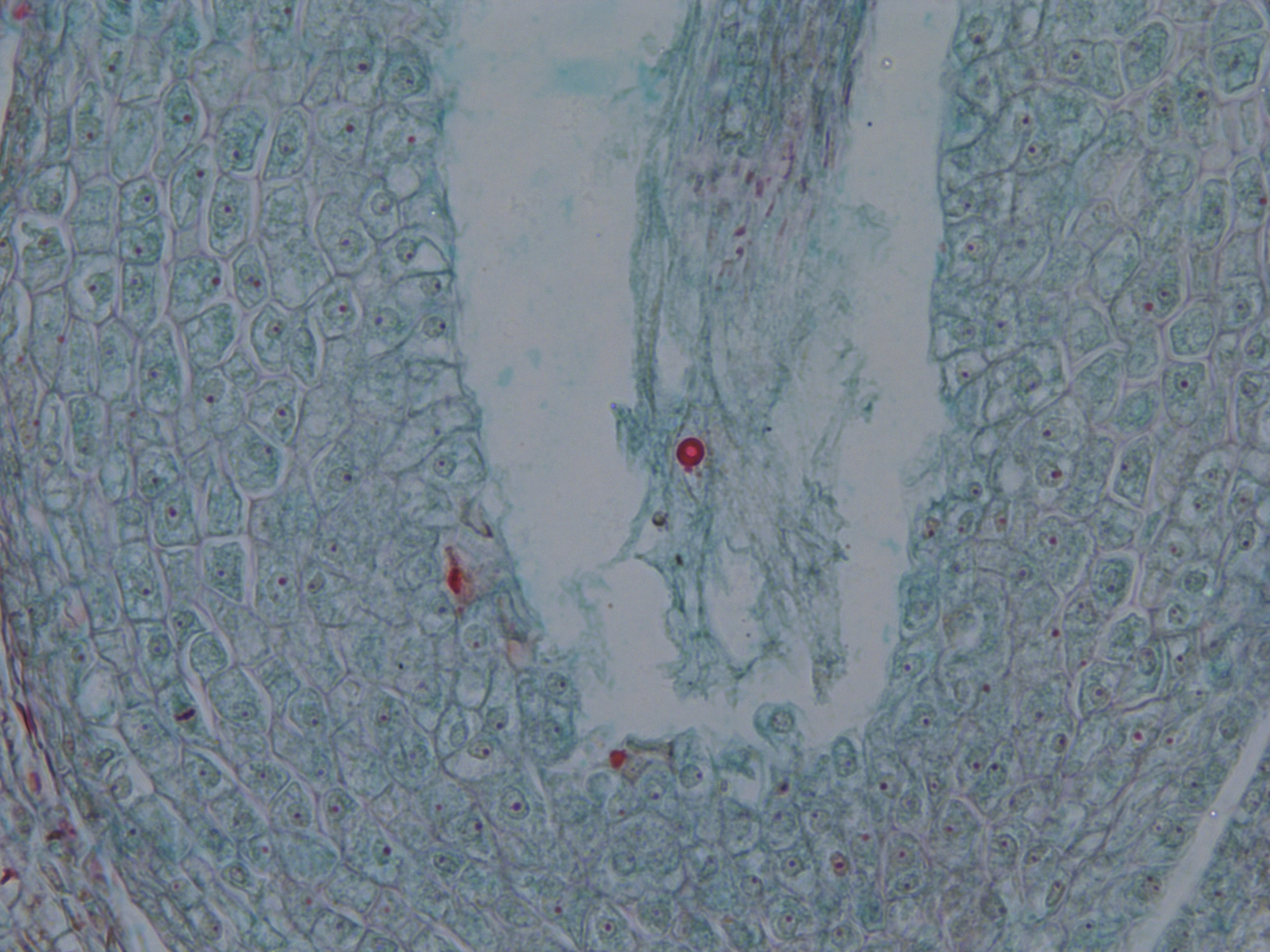

Supplement: Supplemental Information 3 [file peerj-13-18711-s003.zip › 3-m.jpg]

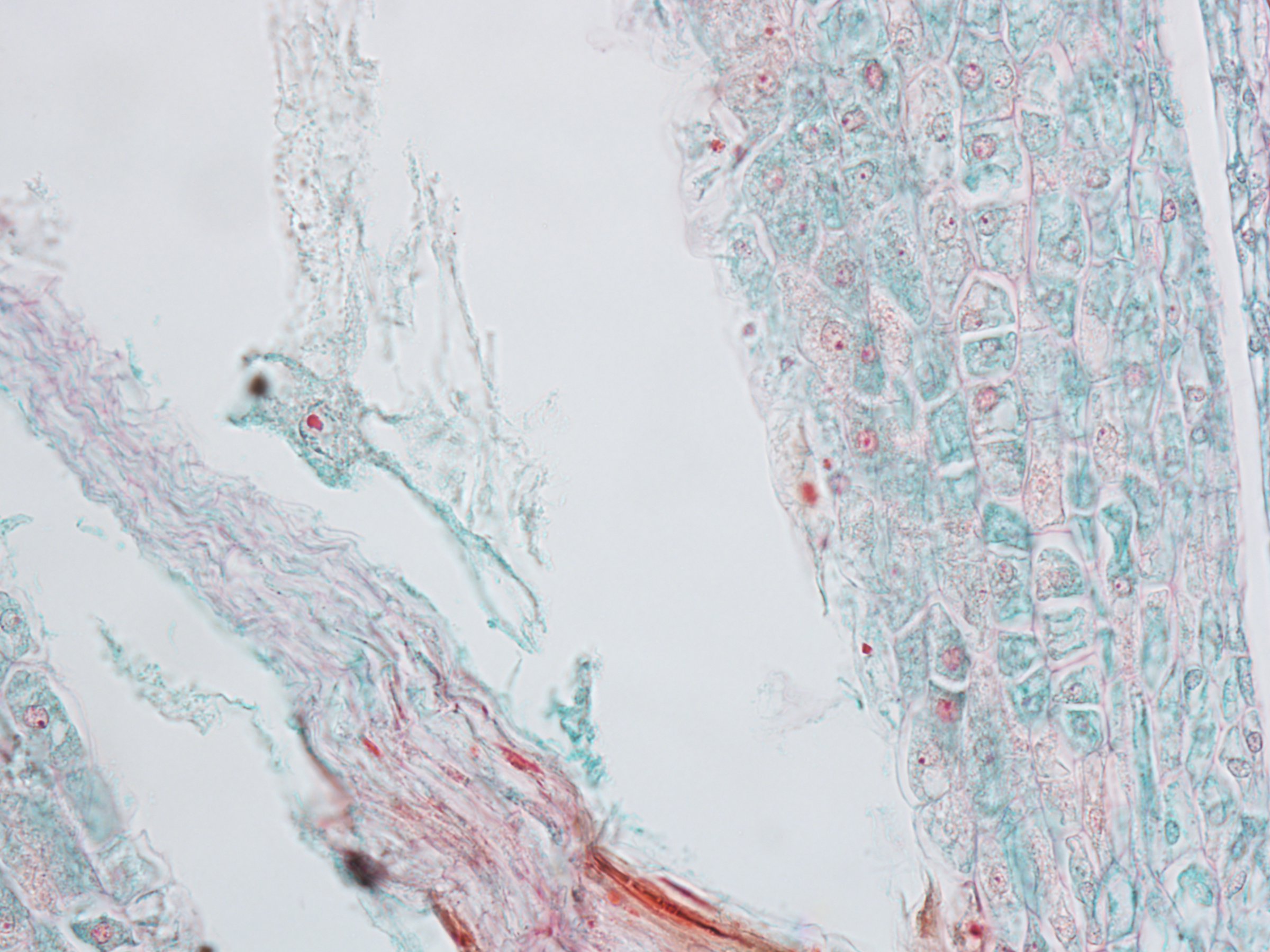

Supplement: Supplemental Information 3 [file peerj-13-18711-s003.zip › 3-n.jpg]

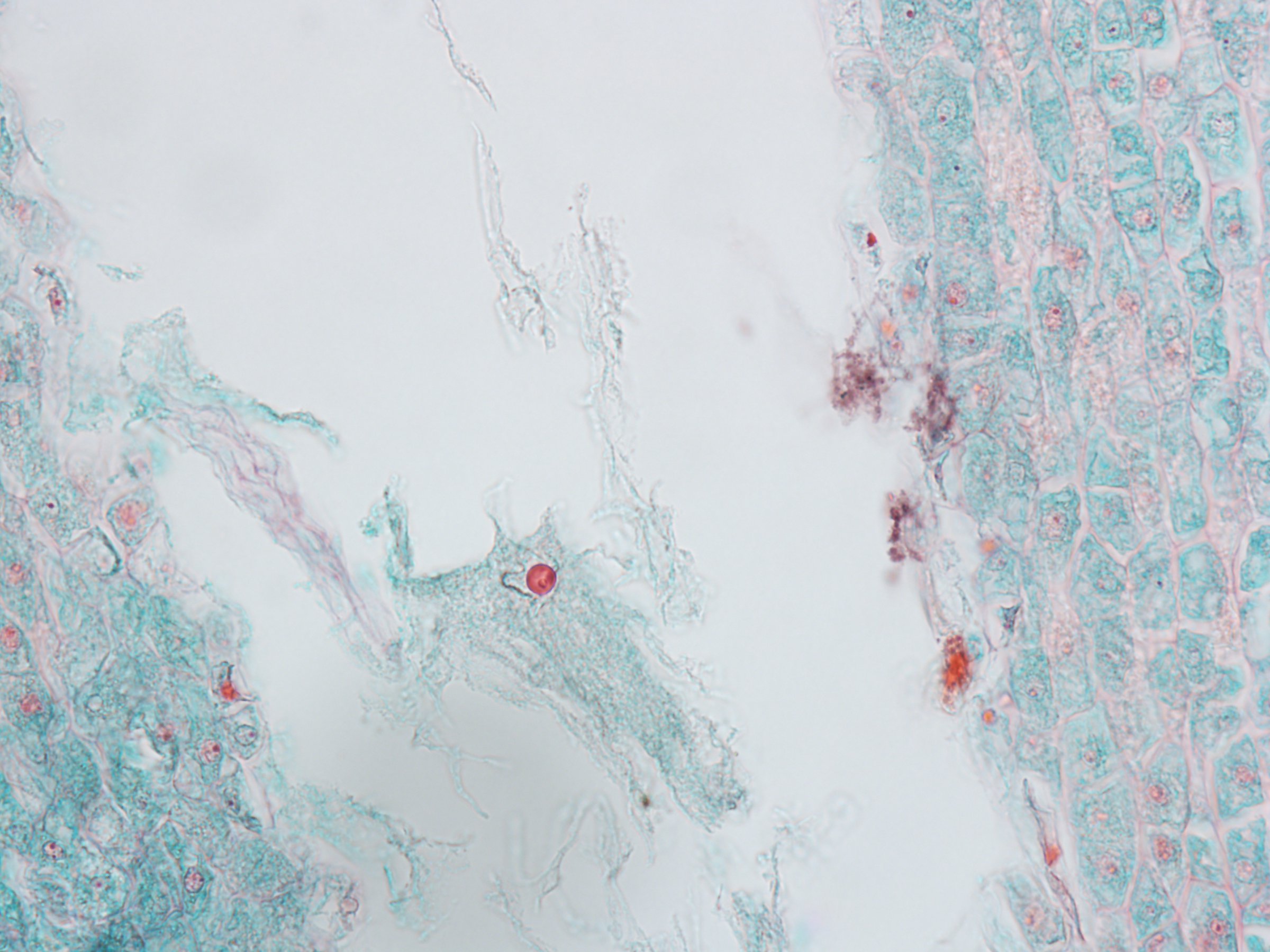

Supplement: Supplemental Information 3 [file peerj-13-18711-s003.zip › 3-o.jpg]

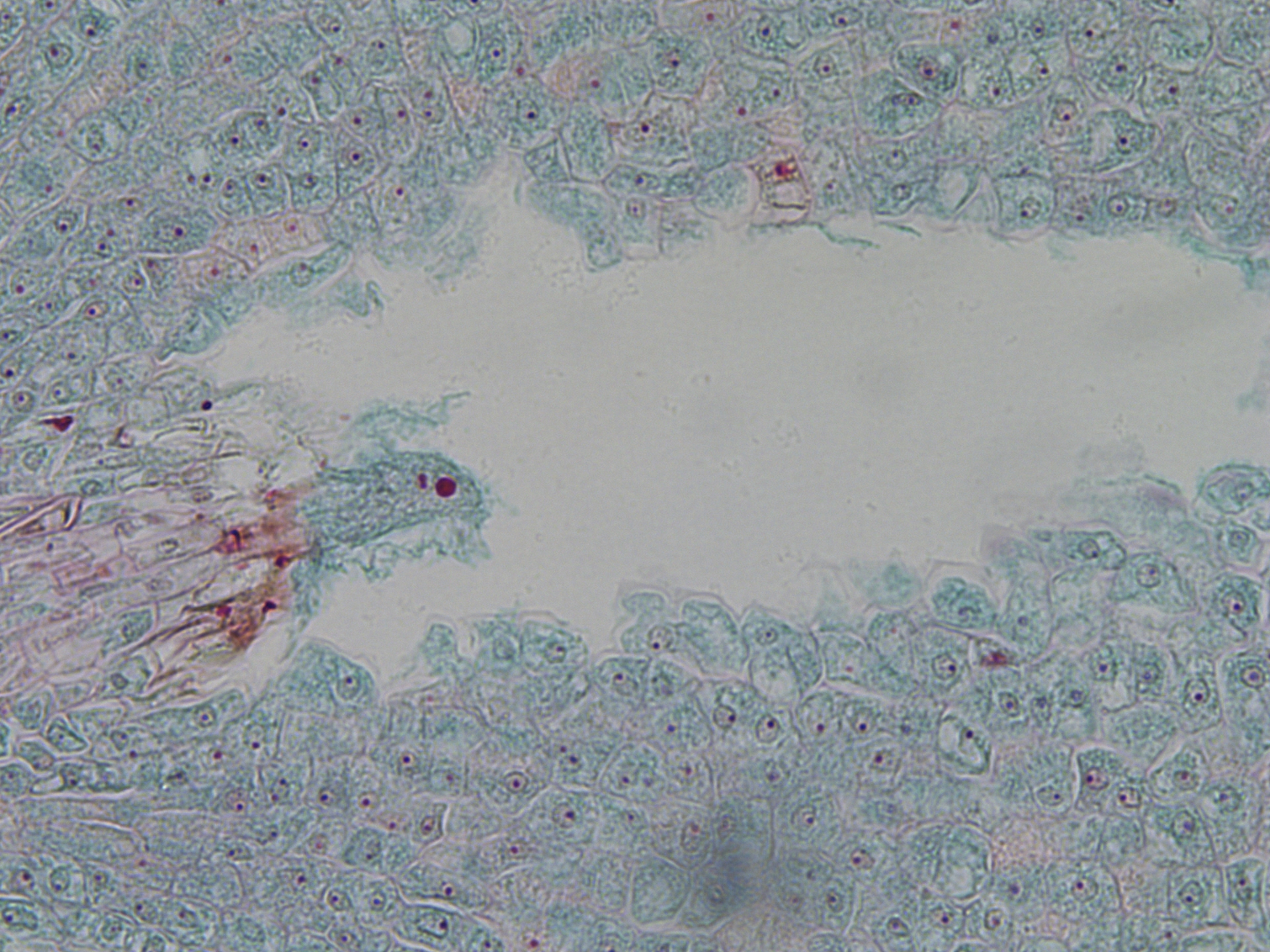

Supplement: Supplemental Information 3 [file peerj-13-18711-s003.zip › 3-p.jpg]

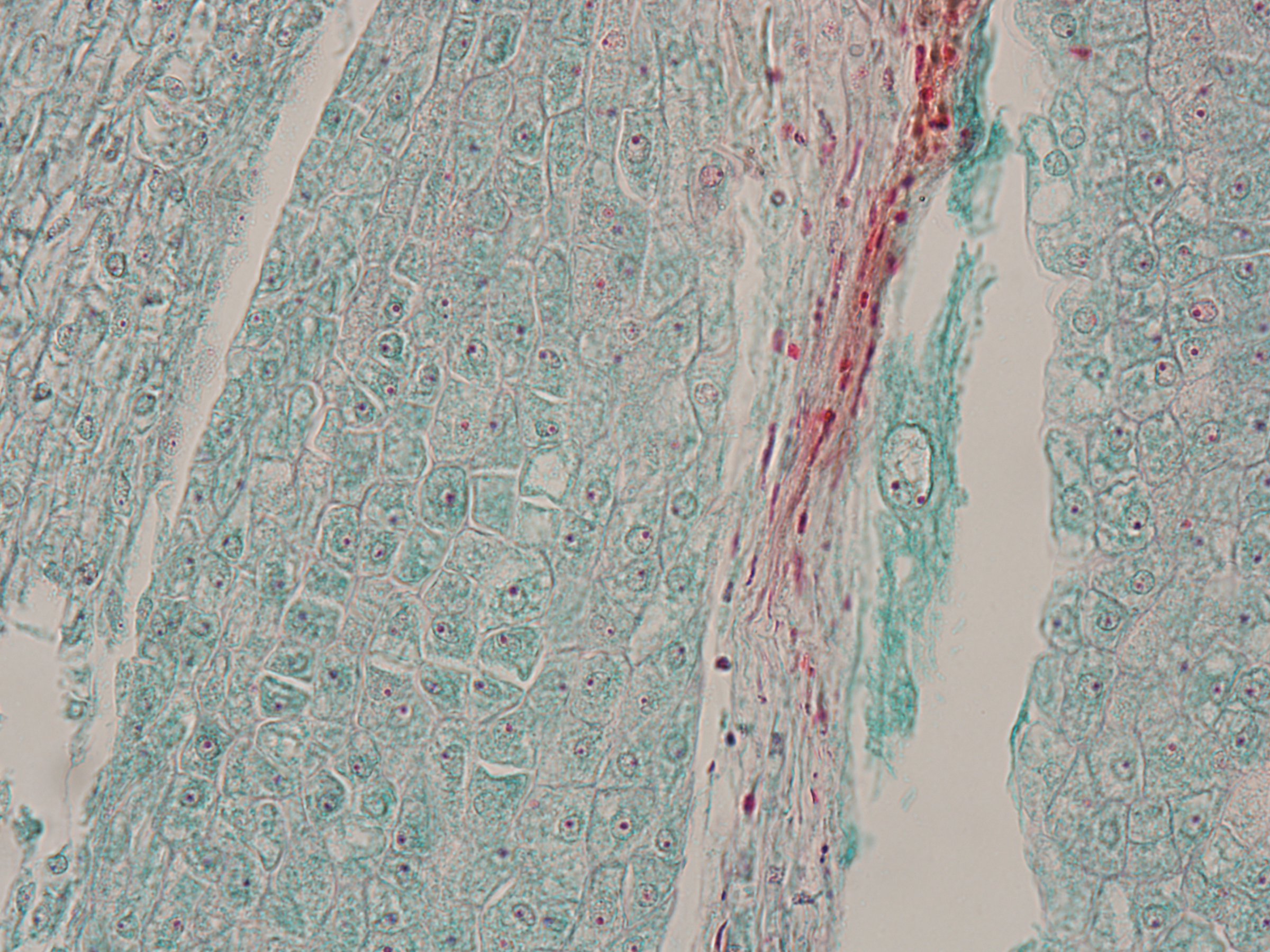

Supplement: Supplemental Information 3 [file peerj-13-18711-s003.zip › 3-q.jpg]

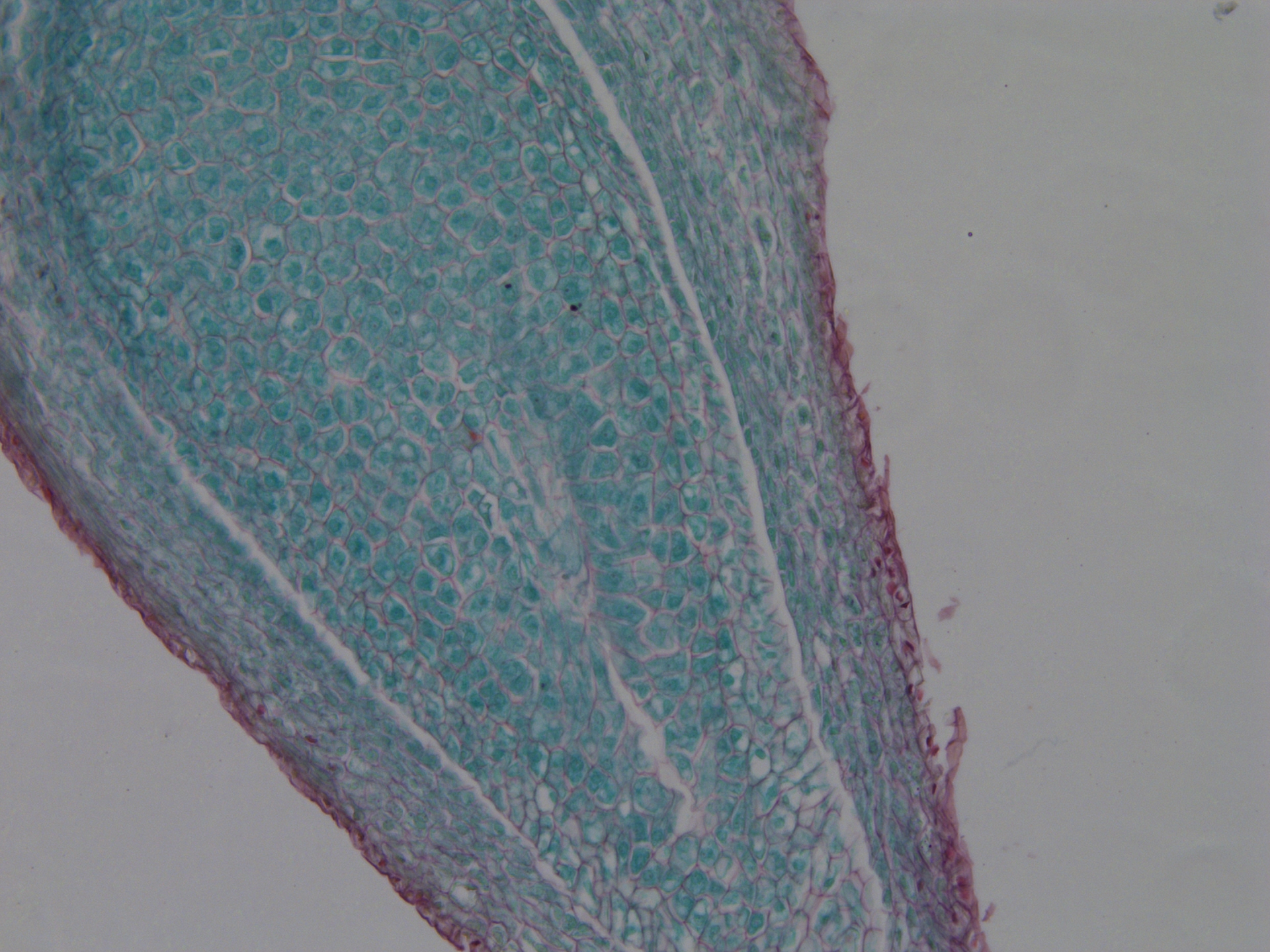

Supplement: Supplemental Information 5 [file peerj-13-18711-s005.zip › Figure 5/6-a.jpg]

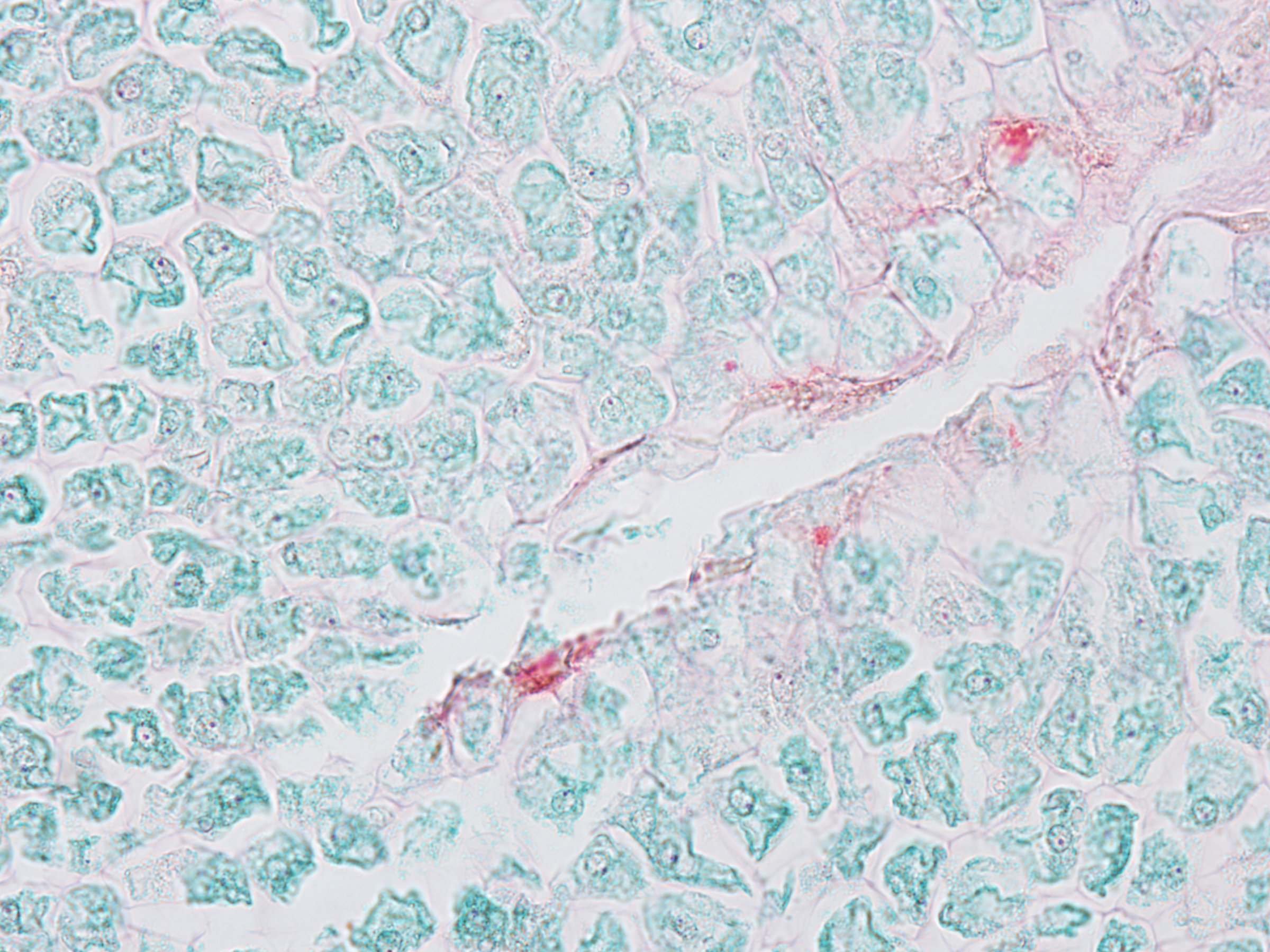

Supplement: Supplemental Information 5 [file peerj-13-18711-s005.zip › Figure 5/6-b.jpg]

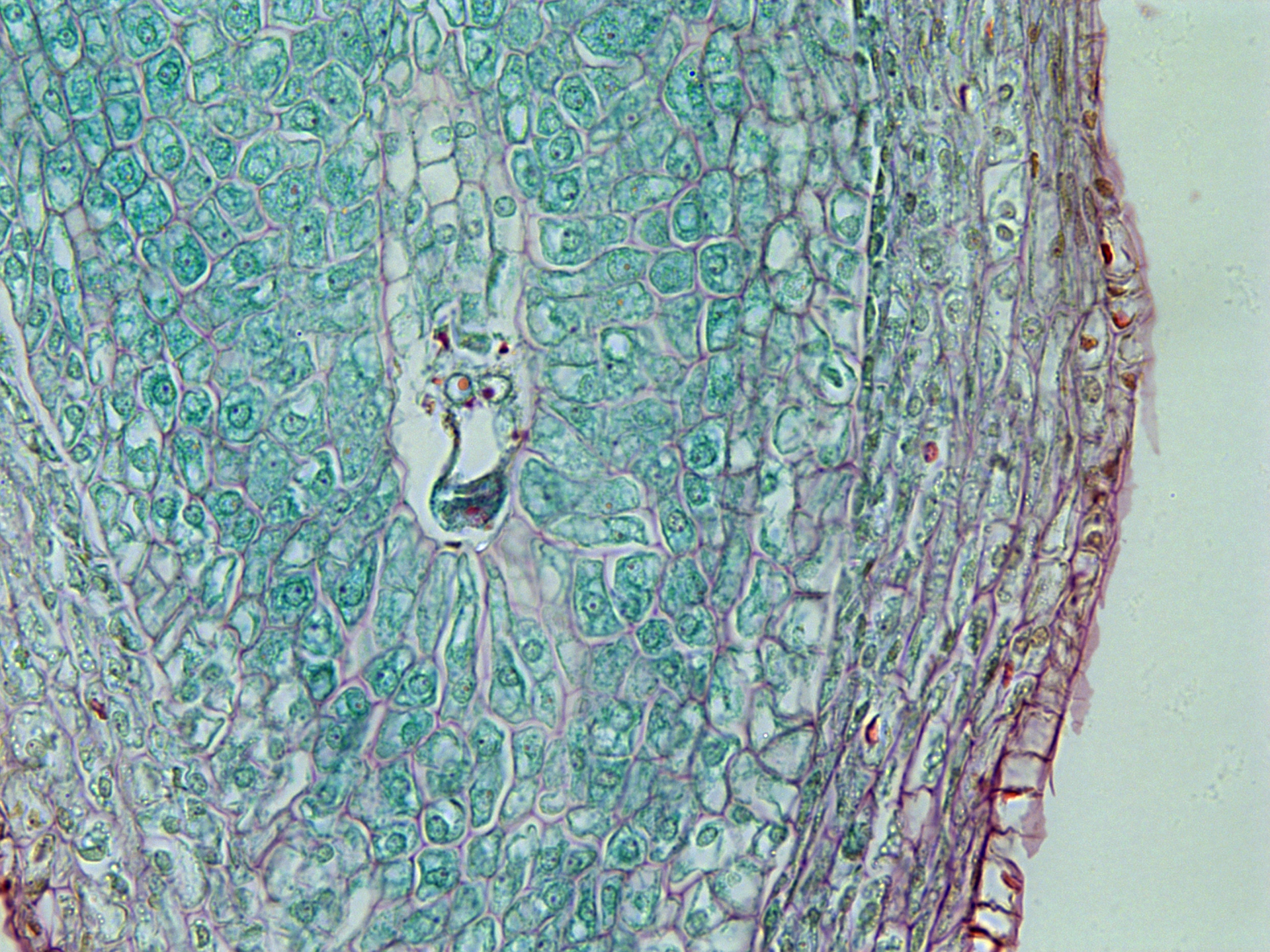

Supplement: Supplemental Information 5 [file peerj-13-18711-s005.zip › Figure 5/6-c.jpg]

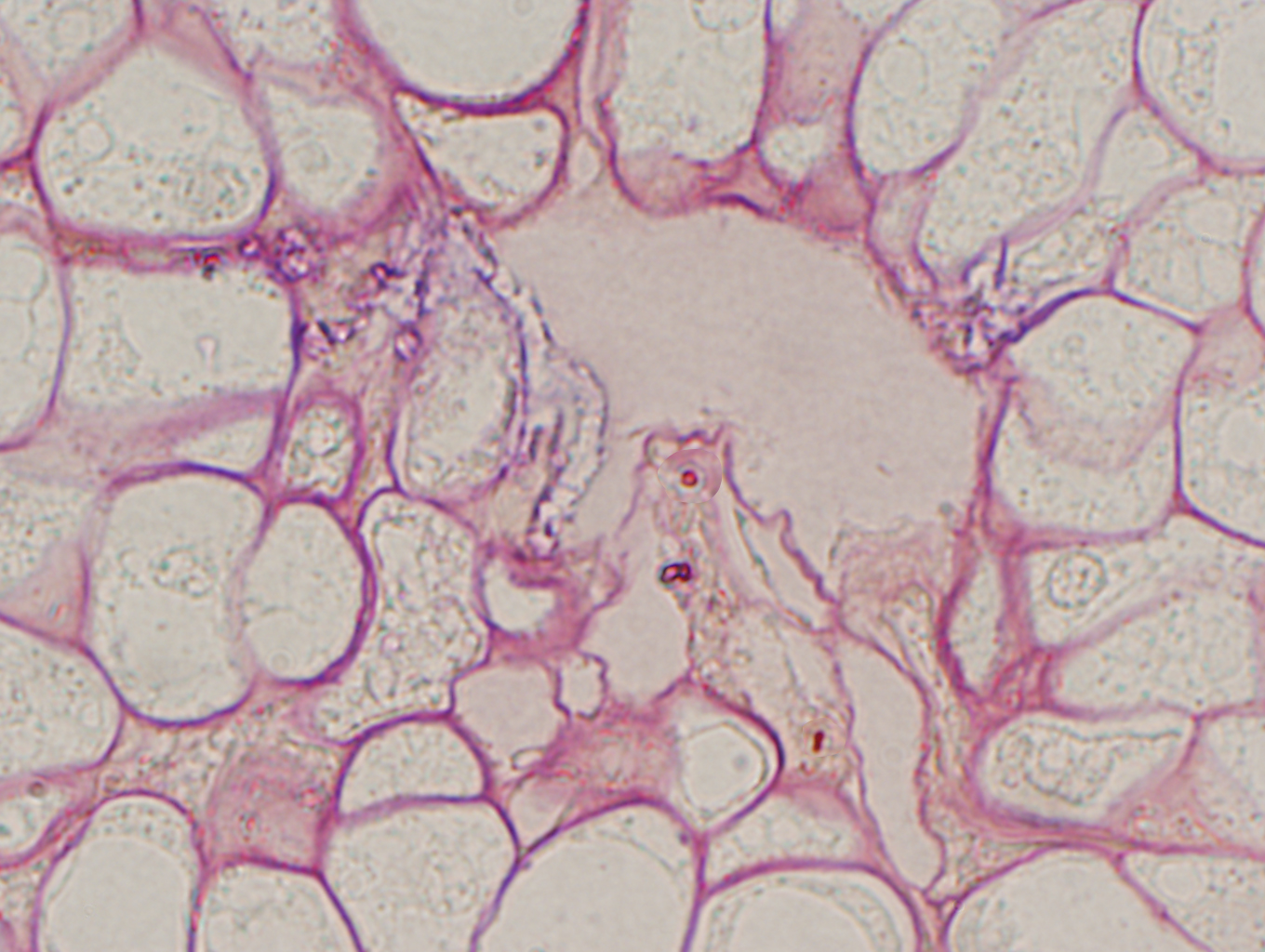

Supplement: Supplemental Information 5 [file peerj-13-18711-s005.zip › Figure 5/6-d.jpg]

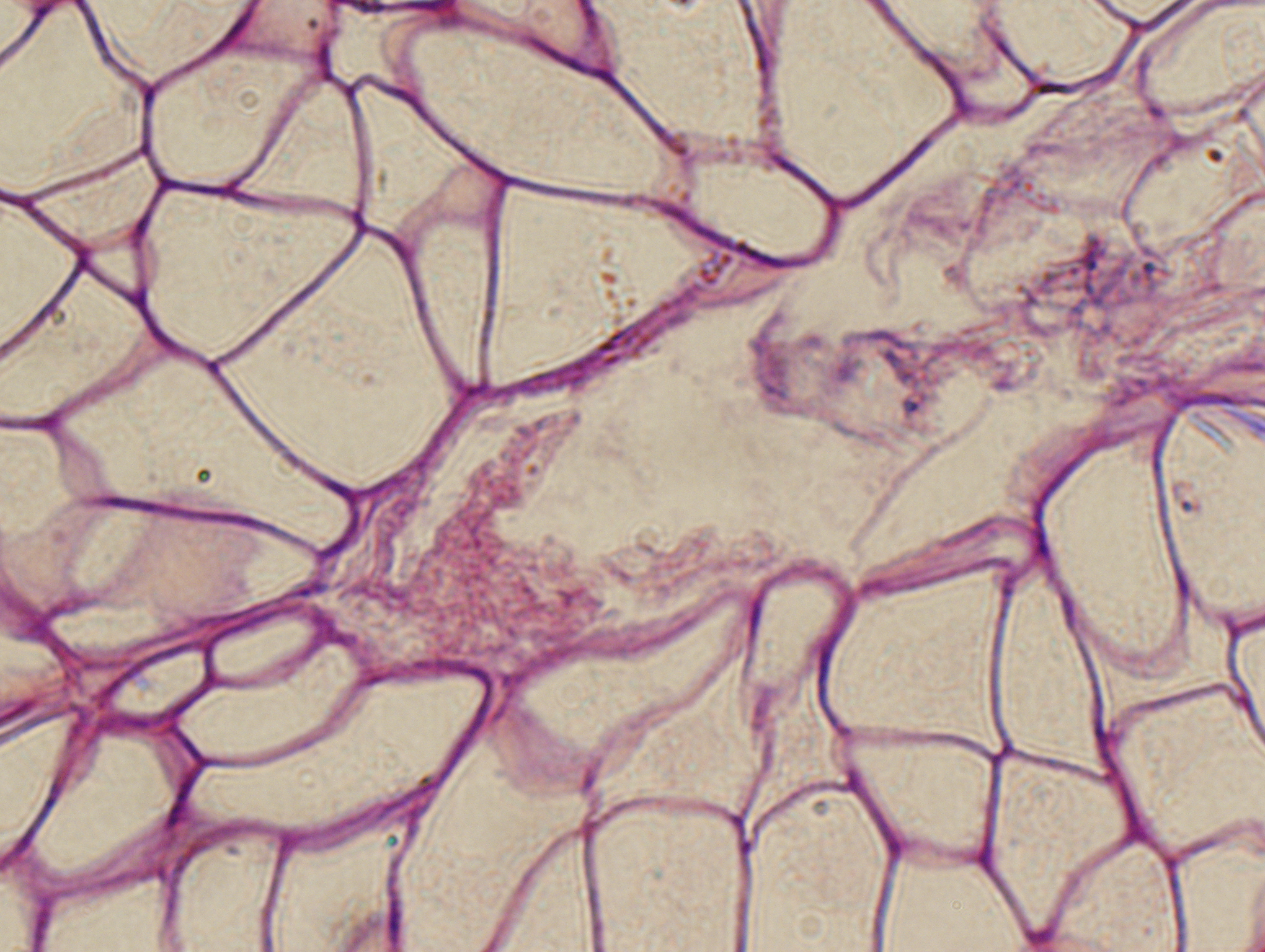

Supplement: Supplemental Information 5 [file peerj-13-18711-s005.zip › Figure 5/6-e.jpg]

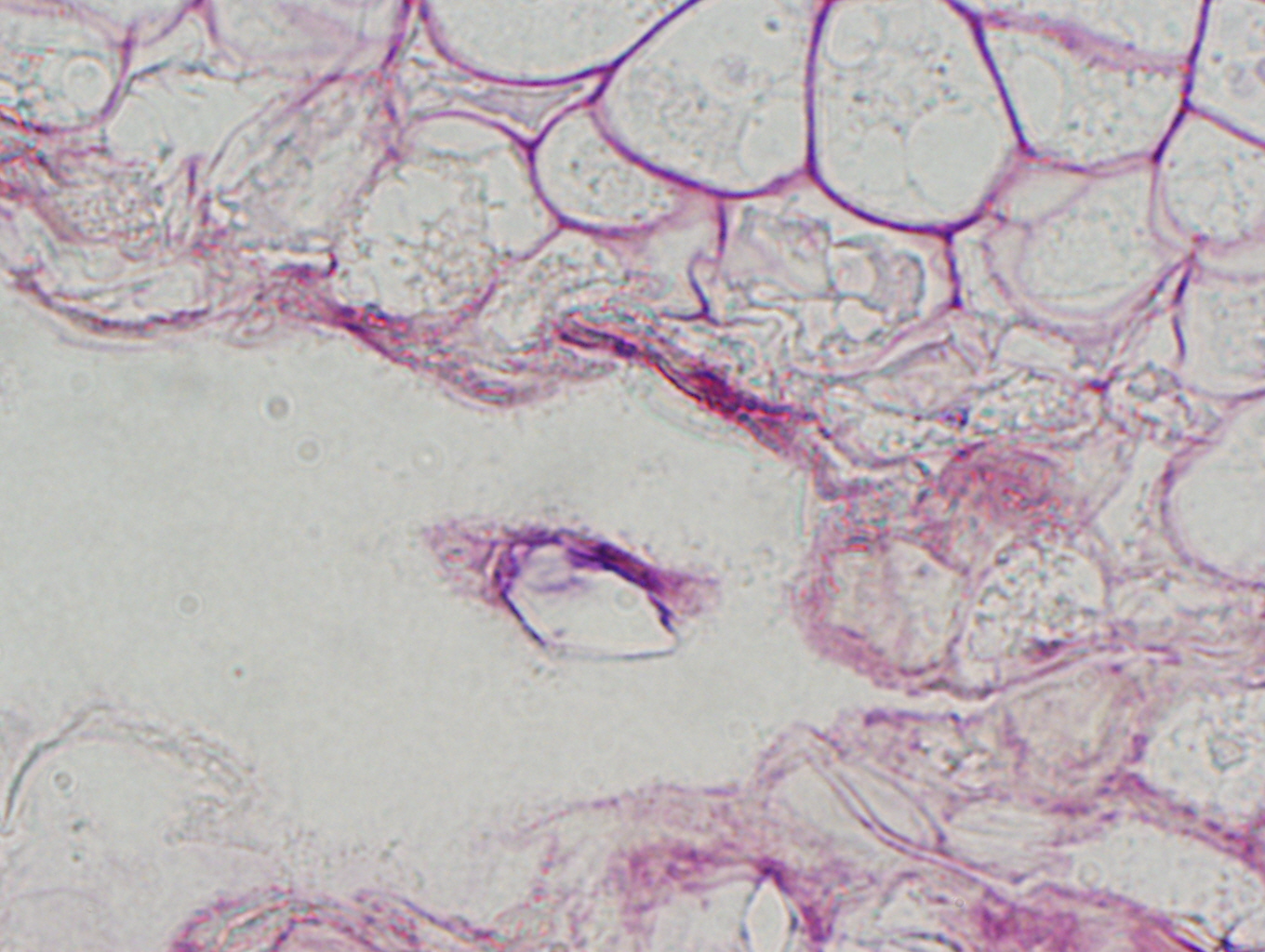

Supplement: Supplemental Information 5 [file peerj-13-18711-s005.zip › Figure 5/6-f.jpg]

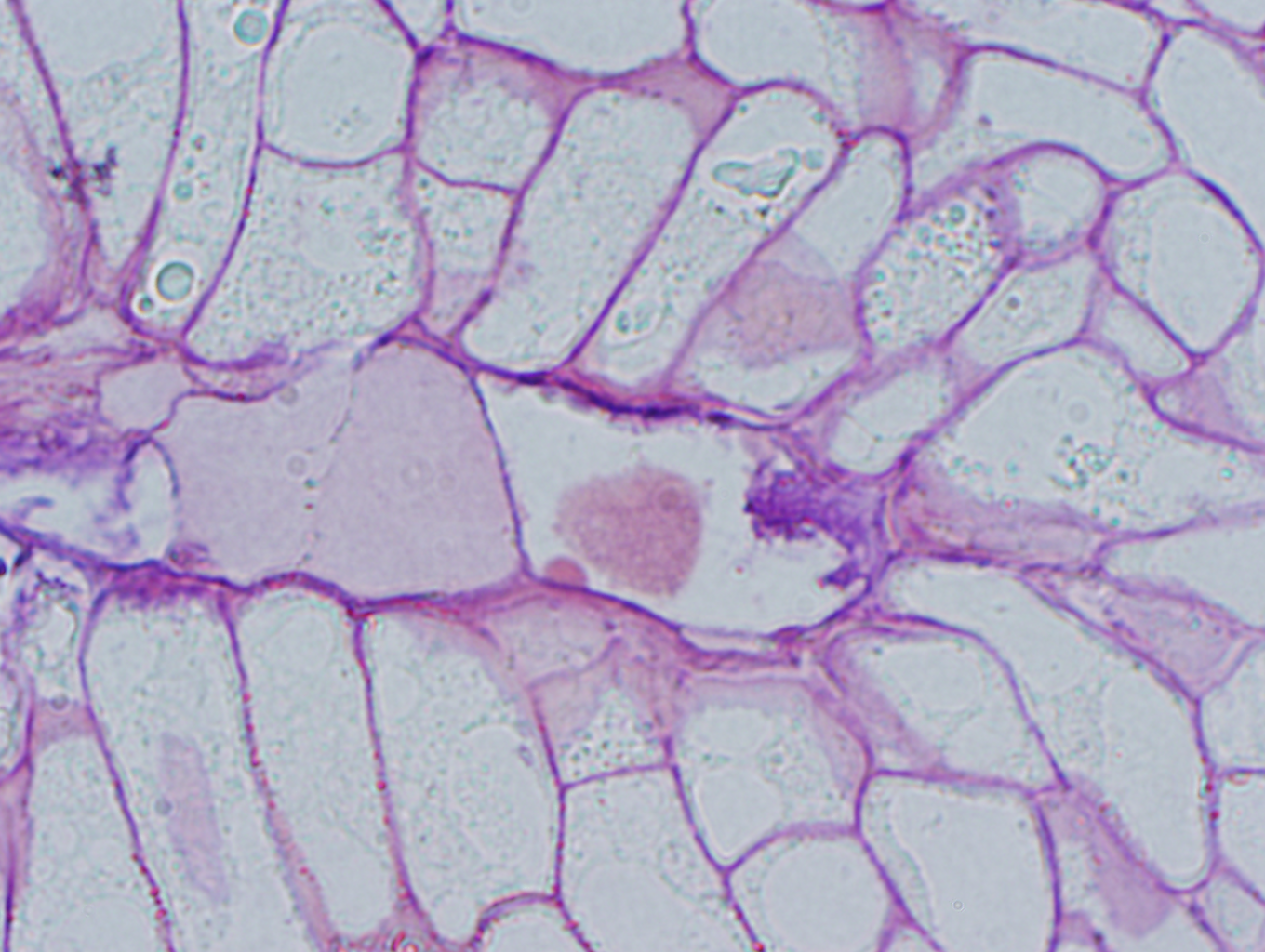

Supplement: Supplemental Information 5 [file peerj-13-18711-s005.zip › Figure 5/6-g.tif]

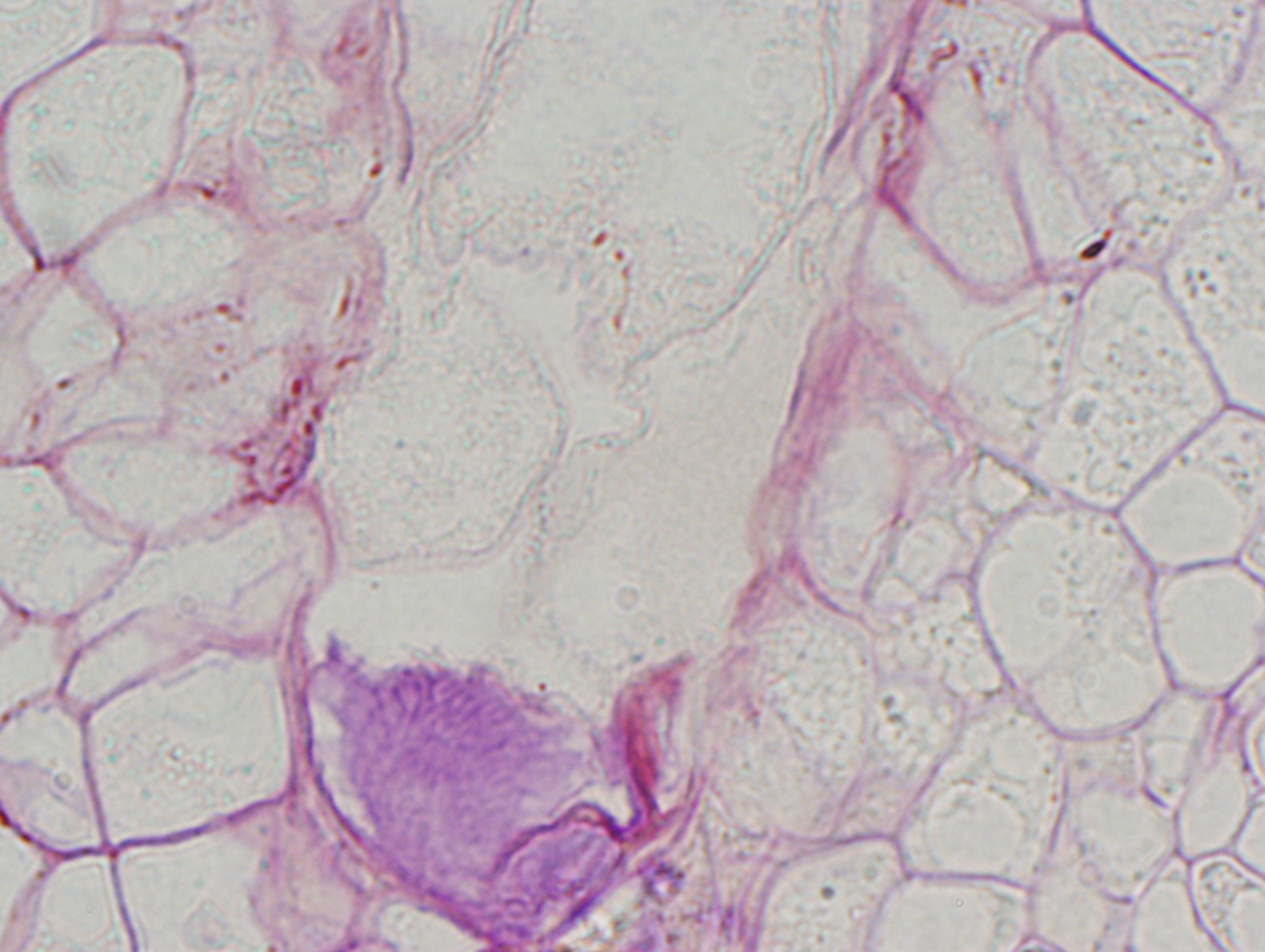

Supplement: Supplemental Information 5 [file peerj-13-18711-s005.zip › Figure 5/6-h.jpg]

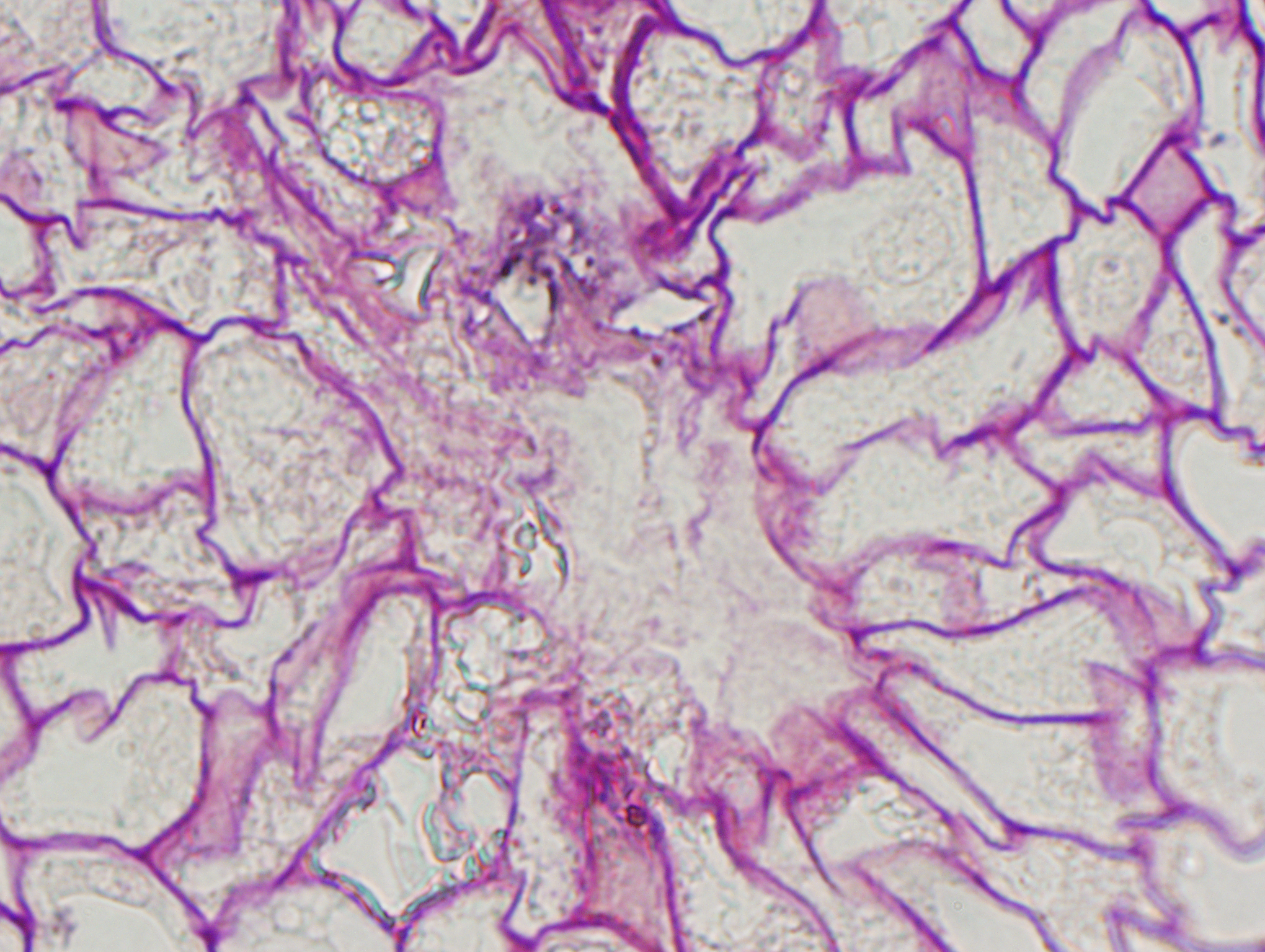

Supplement: Supplemental Information 5 [file peerj-13-18711-s005.zip › Figure 5/6-i.tif]
